# Synergistic In Vitro Effects of Minor Phytocannabinoids and Melatonin Combinations Against Human Glioblastoma Cells

**DOI:** 10.3390/ijms27156774

**Published:** 2026-07-29

**Authors:** Maria Beatrice Morelli, Giorgio Cameli, Martina Giangrossi, Laura Zeppa, Margherita Luongo, Consuelo Amantini, Massimo Nabissi

**Affiliations:** 1 Department of Experimental Medicine and Public Health, School of Pharmacy, University of Camerino, Via Madonna delle Carceri, 62032 Camerino, Italy; giorgio.cameli@unicam.it (G.C.); martina.giangrossi@unicam.it (M.G.); laura.zeppa@unicam.it (L.Z.); 2 “Maria Guarino” Foundation-AMOR No Profit Association, 80078 Pozzuoli, Italy; margluon@gmail.com; 3 School of Medicine and Surgery, Luigi Vanvitelli University of Campania, 81100 Naples, Italy; 4 Department of Experimental Medicine and Public Health, School of Biosciences and Veterinary Medicine, University of Camerino, Via Gentile III da Varano, 62032 Camerino, Italy; consuelo.amantini@unicam.it

**Keywords:** cannabigerol, cannabinol, glioblastoma, melatonin, minor cannabinoids, phytocannabinoids, synergistic cytotoxicity, temozolomide

## Abstract

The prognosis of glioblastoma (GBM) patients remains dismal due to chemoresistance. Repurposing of natural and endogenous compounds, such as the pineal hormone melatonin (MLT) and minor phytocannabinoids like cannabinol (CBN) or cannabigerol (CBG), represents a promising strategy. This study investigates the cytotoxic potential of combining these phytocannabinoids with MLT, evaluating their efficacy both alone and synergistically with temozolomide (TMZ) to overcome drug resistance. To achieve this, cytotoxicity, synergy (Bliss model), and selectivity were evaluated in U87, T98, and U251 GBM lines and normal astrocytes. Mechanisms of damage were characterized via Western blot (γH2AX and PARP-1), flow cytometry using fluorescent dyes/probes (DCFDA, JC-1, MitoBright, BODIPY, PI, and Annexin-V), or the protein marker COX IV and confocal analysis. The results demonstrated that CBN-MLT and CBG-MLT regimens exerted synergistic cytotoxicity while sparing healthy astrocytes. Notably, combining these regimens (U87: MLT 0.3 mg/mL + CBN 25 µM; MLT 0.2 mg/mL + CBG 15 µM. T98: MLT 0.7 mg/mL + CBN 25 µM; MLT 0.6 mg/mL + CBG 30 µM. U251: MLT 0.4 mg/mL + CBN 20 µM; MLT 0.5 mg/mL + CBG 35 µM) with TMZ significantly enhanced chemotherapeutic efficacy, overcoming baseline effects of TMZ in these cell lines. The combinations induced necrotic cell death characterized by severe double-strand DNA damage. This was driven by an early accumulation of intracellular ROS, which triggered mitochondrial depolarization, loss of organelle mass, and lipid peroxidation. CBN combinations consistently triggered more robust biochemical alterations than CBG-based treatments. Taken together, this study provides a strong preclinical basis for utilizing minor cannabinoids combined with MLT in GBM management. Crucially, this co-treatment emerges as a promising approach to potentiate TMZ efficacy, offering a novel and potentially effective therapeutic strategy to counter GBM resilience.

## 1. Introduction

Glioblastoma (GBM) remains the most aggressive, lethal, and histologically malignant primary brain tumor in adults, classified as an Isocitrate Dehydrogenase (IDH)-wildtype, Central Nervous System (CNS) grade 4 astrocytoma by the World Health Organization [1]. The current standard of care, consisting of maximal safe surgical resection followed by radiotherapy and temozolomide (TMZ) chemotherapy, provides only limited clinical benefit [2]. Despite the aggressive therapeutic regimen, the median survival time for GBM patients remains a dismal 12 to 15 months, with a 5-year survival rate of less than 10% [2]. Consequently, there is an urgent clinical need to identify novel, multi-targeted therapeutic strategies that can bypass resistance mechanisms and enhance the efficacy of standard treatments without compounding systemic toxicity.

In recent years, the repurposing of endogenous compounds and natural products has emerged as a promising frontier in neuro-oncology. Among these, the pineal hormone melatonin (MLT) and phytocannabinoids derived from *Cannabis sativa*, most notably Δ^9^-tetrahydrocannabinol (THC) and cannabidiol (CBD), have shown strong individual anti-cancer effects [3,4,5,6,7,8,9,10]. While major cannabinoids like THC and CBD have historically dominated oncology research, minor cannabinoids such as cannabigerol (CBG) and cannabinol (CBN) are becoming more interesting. CBN is a secondary phytocannabinoid formed through the oxidative degradation of THC rather than by direct biosynthesis in *Cannabis sativa*. It has approximatively 10% of the psychoactive potency of THC. Although CBN exhibits low affinity for the canonical cannabinoid receptors, CB1 and CB2, it modulates multiple transient receptor potential (TRP) channels, acting as an agonist of TRPV1–4 and TRPA1 and an antagonist of TRPM8. These interactions are associated with anti-inflammatory, analgesic, antibacterial, and orexigenic activities. Preclinical studies indicate that CBN suppresses pro-inflammatory cytokines (IL-2, IL-4, IL-5, and IL-13), reduces allergen-induced mucus production, exhibits activity against drug-resistant bacteria, and stimulates food intake in rodent models. CBG, generated by decarboxylation of cannabigerolic acid (CBGA), the biosynthetic precursor of both THC and CBD, is a non-psychoactive phytocannabinoid that acts as a weak partial agonist at the CB1 and CB2 receptors. CBG activates TRPV1–4 and TRPA1 channels at low micromolar concentration. CBG also interacts with additional molecular targets, including 5-HT1A and α2-adrenergic receptors, and exerts anti-inflammatory and analgesic effects through CB2-, TRP-, and PPAR-γ-dependent pathways. Furthermore, CBG promotes appetite and food intake in preclinical models, highlighting its therapeutic potential for the management of anorexia [11]. Crucially, phytocannabinoids are capable of inhibiting tumor growth, inducing cancer cell death, and modulating the immune microenvironment [12,13,14,15]. Moreover, they have demonstrated a distinct ability to enhance the effectiveness of conventional therapies and sensitize chemotherapeutic treatments, helping to overcome established resistance mechanisms [6,16,17].

In the context of GBM, MLT has been shown to inhibit GBM cell proliferation by inducing cell cycle arrest, and modulating key oncogenic pathways [3,18,19,20]. Furthermore, MLT acts as an antioxidant, protecting healthy neural tissue from therapy-induced toxicity while selectively promoting cell death mechanisms within GBM cells [3,4]. While both MLT and phytocannabinoids have demonstrated independent efficacy in slowing GBM progression, single-agent therapies often fall short due to the redundant, compensatory signaling pathways inherent to GBM. The rationale for combining MLT and phytocannabinoids lies in their distinct primary receptor-mediated mechanisms of action, specifically through MT1/MT2 receptors for MLT, and CB1/CB2 or TRPV channels for phytocannabinoids, and their shared capacity to cross the blood–brain barrier (BBB) [21,22,23]. Thus, targeting different upstream receptors, they may enhance their therapeutic potential. Emerging evidence, including a few case reports documenting the off-label co-administration of medical cannabis and high-dose MLT, suggests that the simultaneous administration of these compounds may produce a powerful cooperative effect [24,25,26,27,28,29]. Additionally, both MLT and phytocannabinoids have well-documented neuroprotective profiles in healthy tissues, offering the dual benefit of selectively attacking the tumor while shielding adjacent healthy cells from treatment-induced damage [30,31,32,33,34].

This study aimed to investigate the in vitro efficacy of combining MLT with CBN or CBG in human GBM models. We evaluated the impact of this combinatorial approach on cell viability, cytotoxic synergy, and selectivity profiles using a panel of genetically different GBM cell lines and healthy human astrocytes. Furthermore, we investigated the mechanisms of cell death and the underlying damage pathways, providing robust biochemical validation for a promising multi-target therapeutic strategy against GBM.

## 2. Results

### 2.1. Synergistic Effects of MLT-CBN and MLT-CBG Combinations in GBM Cell Lines

**Figure 1:**
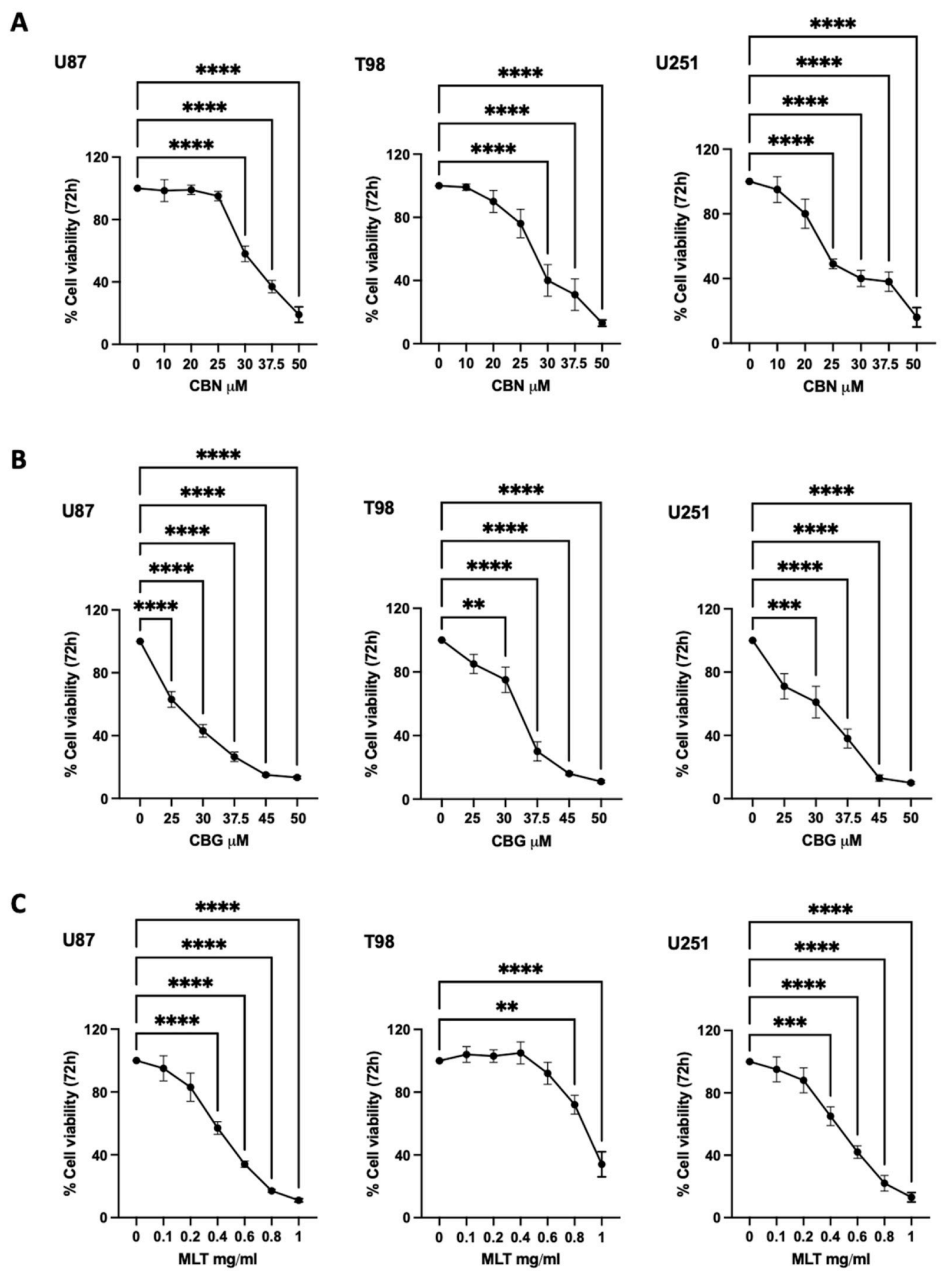
Dose-dependent effects of MLT, CBG, and CBN on human GBM cell viability. Cell viability of U87 (**A**), T98 (**B**), and U251 (**C**) glioma cells was evaluated by 3-[4,5-dimethylthiazol-2-yl]-2,5 diphenyl tetrazolium bromide MTT assay after 72 h of treatment with increasing concentrations of CBN (0–50 µM), CBG (0–50 µM), or MLT (0–1 mg/mL) administered as single-agent treatments. The 100% viability baseline corresponds to the vehicle-treated control cells. Data are expressed as the mean ± standard deviation (SD) of three independent experiments. Statistical analysis was performed using one-way ANOVA followed by Dunnett’s post hoc test ** *p* < 0.01, *** *p* < 0.001, and **** *p* < 0.0001 vs. control (0).

To establish the inhibitory effects of the single agents on cell viability, U87, T98, and U251 GBM cell lines were treated with increasing concentrations of CBN (0–50 µM), CBG (0–50 µM), or MLT (0–1 mg/mL) for 72 h (Figure 1). The selected concentration ranges were sufficient to capture a comprehensive dose-dependent toxicity, reaching a reduction in cell viability down to approximately 10% at the highest doses for all agents. Higher concentrations were not further evaluated as the maximum cytotoxic effect was already successfully achieved, and the calculated IC_50_ values provided a robust baseline to design the subsequent low-dose synergistic combinations.

As shown in Figure 1A, single-agent CBN induced a significant dose-dependent reduction in cell viability across all three cell lines. While lower concentrations (up to 20 µM) showed minimal-to-moderate effects, a drop in viability was observed at higher doses, with 50 µM CBN decreasing cell survival below 20% in U87, T98, and U251 cells (*p* < 0.0001 vs. control), yielding calculated IC_50_ values of 34 ± 0.05 µM for U87, 30 ± 0.33 µM for T98, and 28 ± 0.85 µM for U251 cells.

A similar dose–response pattern was observed for CBG with IC_50_ values of 28 ± 0.01 µM, 34 ± 2.41 µM, and 32 ± 0.01 µM for U87, T98, and U251, respectively (Figure 1B).

Regarding MLT, to rationalize its use, we first verified the endogenous presence of MLT receptors (MEL A/B) in the selected human GBM cell lines via Western blot analysis. As shown in Figure S1, all three cell lines exhibited basal expression of MLT receptors with MIA PaCa-2 pancreatic ductal adenocarcinoma cells serving as a positive control [24]. Following this validation, we evaluated the in vitro efficacy of MLT (Figure 1C). It exerted a less pronounced cytotoxic profile at lower tested doses, particularly in T98 cells, where viability remained close to 100% up to 0.6 mg/mL (equivalent to a high concentration of 2.6 mM). At higher concentrations, MLT induced a statistically significant, dose-dependent reduction in cell viability, yielding IC_50_ values of 0.43 ± 0.01 mg/mL (equivalent to 1.86 ± 0.05 mM) for U87, 0.91 ± 0.02 mg/mL (equivalent to 3.92 ± 0.1 mM) for T98, and 0.49 ± 0.02 mg/mL (equivalent to 2.15 ± 0.1 mM) for U251 cells. These results indicate that its intrinsic inhibitory effects become apparent only at doses exceeding physiological levels and conventional clinical therapeutic ranges.

Taken together, these data provided the necessary concentration ranges to further investigate potential synergistic interactions and selectivity profiles in subsequent combination assays.

**Figure 2:**
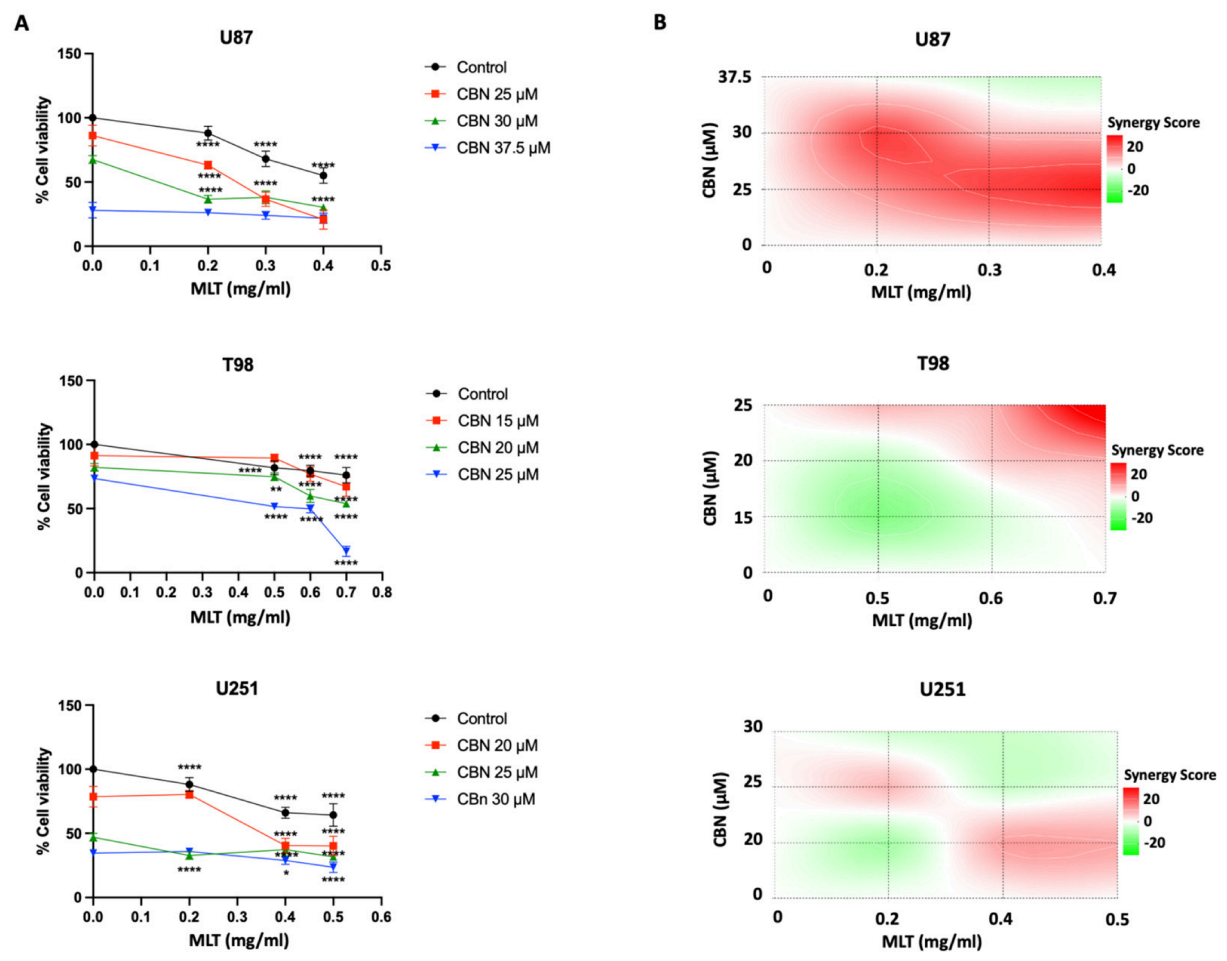
In vitro cell viability assay and synergistic interaction maps of CBN-MLT combinations in GBM cell lines. (**A**) Dose–response curves of U87, T98, and U251 cells treated with three concentrations of CBN, either alone or in combination with increasing doses of MLT. Data are presented as absolute cell viability (%) relative to untreated cells (set at 100%). Data are expressed as the mean ± SD of three independent experiments. Statistical significance was determined using two-way ANOVA followed by Dunnett’s post hoc test (* *p* < 0.05, ** *p* < 0.01, **** *p* < 0.0001 vs. control). (**B**) 2D synergy landscapes calculated using the Bliss independence model for CBN + MLT. The color scale indicates the synergy score: red regions represent synergistic cytotoxicity (score > 10), white regions indicate additive effects (−10 ≤ score ≤ 10), and green regions represent antagonism (score < −10).

**Figure 3:**
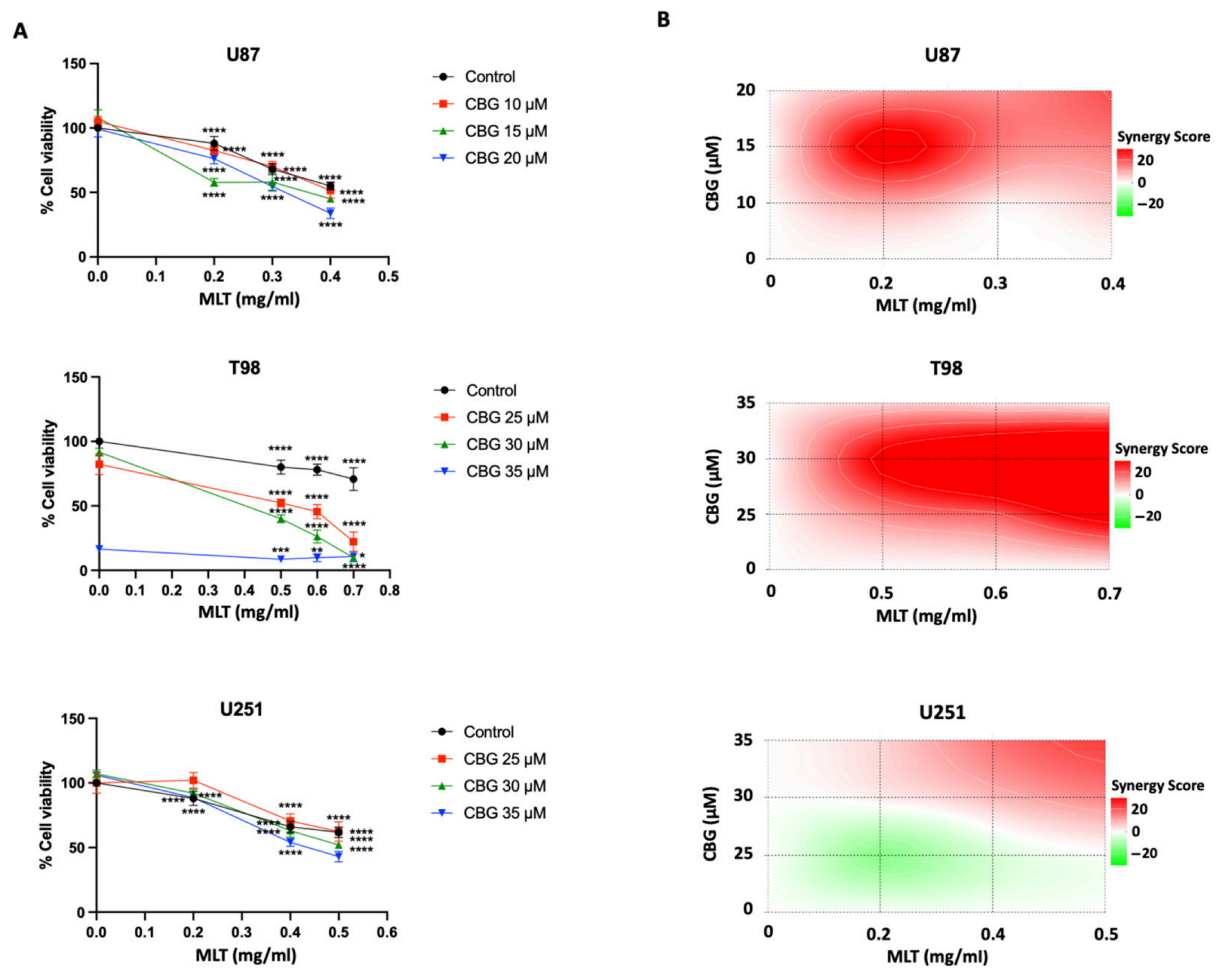
In vitro cell viability assay and synergistic interaction maps of CBG-MLT combinations in GBM cell lines. (**A**) Dose–response curves of U87, T98, and U251 cells treated with three concentrations of CBG, either alone or in combination with increasing doses of MLT. Data are presented as absolute cell viability (%) relative to untreated cells (set at 100%). Data are expressed as the mean ± SD of three independent experiments. Statistical significance was determined using two-way ANOVA followed by Dunnett’s post hoc test (* *p* < 0.05, ** *p* < 0.01, *** *p* < 0.001, **** *p* < 0.0001 vs. control). (**B**) 2D synergy landscapes calculated using the Bliss independence model for CBG + MLT. The color scale indicates the synergy score: red regions represent synergistic cytotoxicity (score > 10), white regions indicate additive effects (−10 ≤ score ≤ 10), and green regions represent antagonism (score < −10).

To explore potential therapeutic advantages, we next evaluated the combined effects of CBG or CBN with MLT on U87, T98, and U251 cells (Figure 2 and Figure 3). Combined treatment with increasing doses of CBN and MLT produced a dose-dependent decrease in cell viability, demonstrating a significantly enhanced cytotoxic effect compared with the respective single treatments in all three cell lines (Figure 2A). To characterize the nature of these interactions, 2D synergy landscapes were generated using the Bliss independence model (Figure 2B). This analysis revealed a strong synergistic effect (red regions) across intermediate-to-high concentration ranges. Notably, the CBN-MLT combination displayed pronounced synergy in U87 cells at MLT concentrations of 0.2–0.4 mg/mL and in T98 cells at 0.6–0.7 mg/mL.

Similarly, combined treatment with CBG and MLT resulted in a significant reduction in GBM cell viability (Figure 3A). Bliss synergy analysis (Figure 3B) further confirmed a robust and widespread synergistic interaction for the CBG–MLT combination, particularly in U87 and T98 cells, where high positive synergy scores predominated across the treatment matrix at MLT concentrations of 0.2–0.4 mg/mL and 0.5–0.7 mg/mL, respectively. Regarding U251 cells, both combinations exhibited a more localized and concentration-dependent synergistic profile; specifically, the CBN-MLT matrix (Figure 2B) displayed a restricted synergistic area at intermediate CBN concentrations, while the CBG-MLT combination (Figure 3B) showed effective synergy predominantly at the highest concentration ranges. In contrast, antagonistic or weakly additive effects (green/white regions) were observed only at the lowest concentration ranges, but also at the highest tested doses of cannabinoids in U87 and U251 cells, indicating a concentration-dependent limit to the synergistic interaction. Based on these maps, optimal combinations were selected for subsequent experiments (U87: MLT 0.3 mg/mL and CBN 25 µM; MLT 0.2 mg/mL and CBG 15 µM. T98: MLT 0.7 mg/mL and CBN 25 µM; MLT 0.6 mg/mL and CBG 30 µM. U251: MLT 0.4 mg/mL and CBN 20 µM; MLT 0.5 mg/mL and CBG 35 µM).

**Figure 4:**
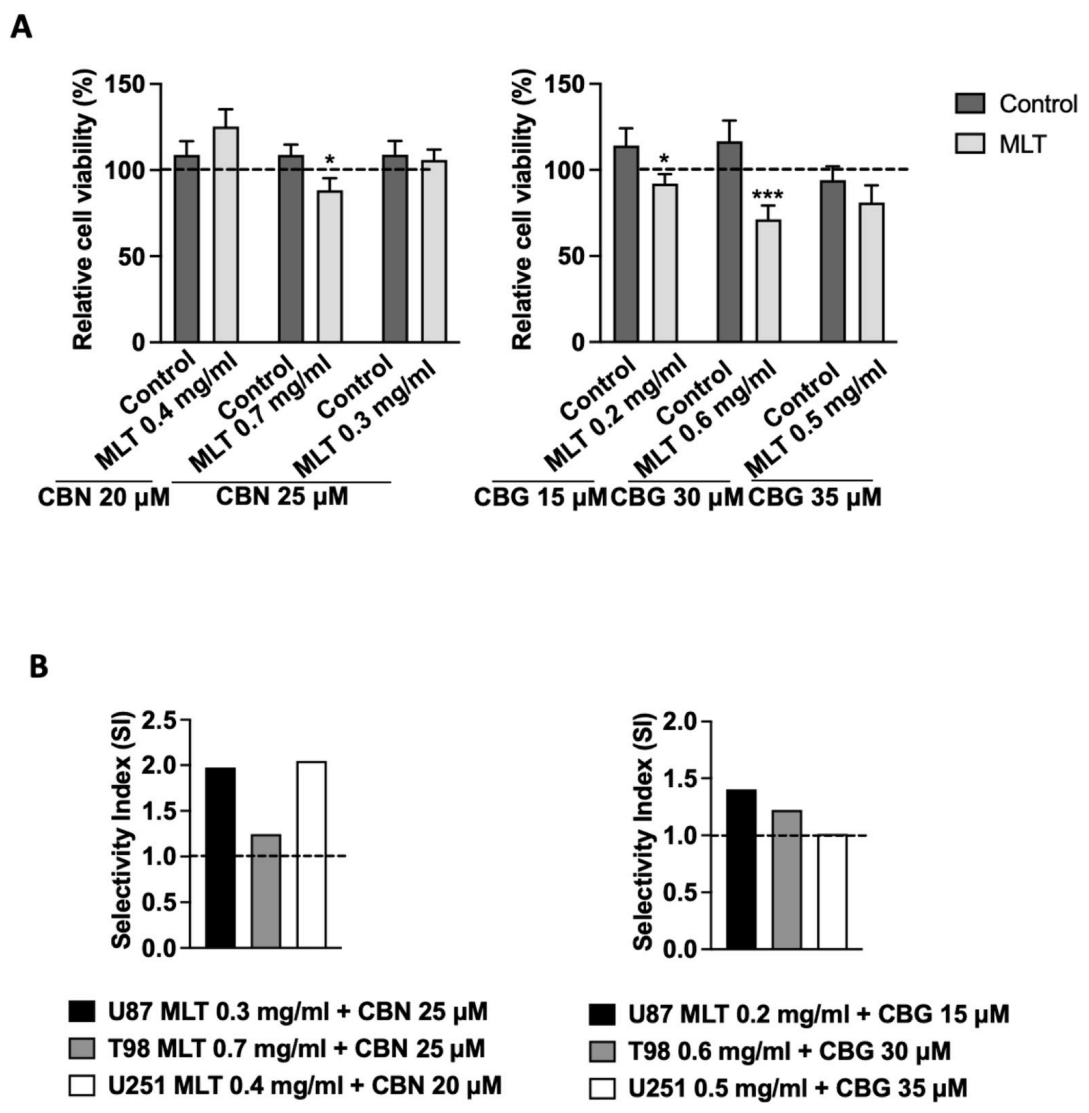
Relative cell viability of healthy AST-1 normal human astrocyte cells and Net Selectivity Indices (SIs) of cannabinoid–MLT combinations. (**A**) Viability of AST-1 cells treated with CBN or CBG in the absence (control) or presence of MLT. Data are expressed as relative cell viability (%) normalized to the respective MLT-alone at each concentration (set at 100%, dashed line). Data are presented as the mean ± SD. Statistical analysis was performed using two-way ANOVA followed by Šidák’s post hoc test (* *p* < 0.05, *** *p* < 0.001 vs. control). (**B**) Net Selectivity Index (SI) calculated for U87, T98, and U251 GBM lines relative to AST-1 cells; the dashed line at SI = 1.0 represents the threshold of non-selectivity (values > 1.0 indicate preferential cytotoxicity toward cancer cells).

To assess the potential impact of these synergistic combinations on non-malignant cells, their effects were evaluated in the AST-1 normal human astrocytes cell line, and the corresponding Net Selectivity Indices (SIs) were calculated to determine the degree of tumor selectivity (Figure 4). In AST-1 cells, viability was expressed as relative cell viability (%) normalized to the respective MLT-alone baselines to isolate the net impact of the cannabinoids. As shown in Figure 4A, the majority of the tested combinations demonstrated a highly favorable safety profile, maintaining normal astrocyte viability close to or above 80–90%. While a statistically significant reduction in viability was noted in a few specific conditions, CBN 25 µM + MLT 0.7 mg/mL (* *p* < 0.05), CBG 15 µM + MLT 0.2 mg/mL (* *p* < 0.05), and CBG 30 µM + MLT 0.6 mg/mL (*** *p* < 0.001), the overall cell survival remained robust, and no critical synergistic toxicity was observed in the remaining combinations.

To quantify the specific cytotoxic effects, the Net Selectivity Index (SI) was calculated for the synergistic clusters identified in each GBM line (Figure 4B). For CBN-MLT combinations, a good selectivity profile was achieved in U251 cells (SI = 2.05) treated with 0.4 mg/mL MLT + 20 µM CBN and U87 cells (SI = 1.97) treated with 0.3 mg/mL MLT + 25 µM CBN. Similarly, CBG-MLT combinations displayed clear therapeutic relevance, with the highest selectivity observed in U87 cells, reaching an SI value of 1.4 (0.2 mg/mL MLT + 15 µM CBG). Taken together, these data indicate that the cytotoxic mechanism of these combinations is safely and preferentially directed toward the GBM phenotype, preserving an encouraging safety margin in healthy brain cells.

### 2.2. Combined Treatments Induce Necrotic Cell Death and DNA Damage

**Figure 5:**
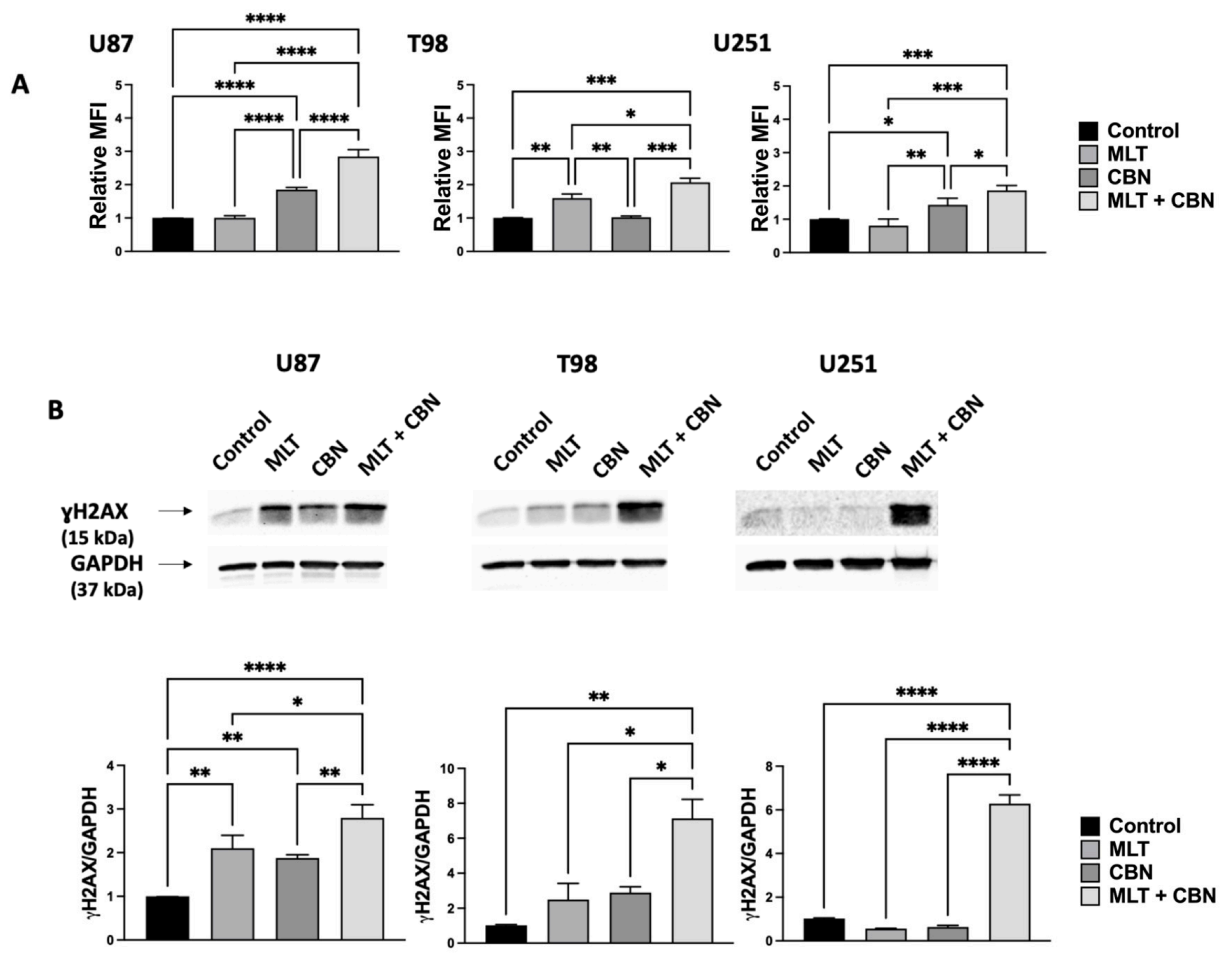
Evaluation of cell death and DNA damage in GBM cell lines following MLT and CBN, alone or in combination. GBM cell lines were treated with MLT or/and CBN for 24 h (U87: MLT 0.3 mg/mL and CBN 25 µM; T98: MLT 0.7 mg/mL and CBN 25 µM; U251: MLT 0.4 mg/mL and CBN 20 µM). (**A**) Cell death was assessed via PI staining and flow cytometry. Bar graphs represent the relative MFI, expressed as fold change relative to the untreated control for U87, T98, and U251 lines under single or combined MLT and CBN exposure. In all bar graphs, the control group was normalized to 1. Data are presented as the mean ± SD of at least three independent biological replicates. (**B**) Western blot analysis (top) and corresponding densitometric quantification (bottom) of γH2AX protein levels relative to Glyceraldehyde-3-Phosphate Dehydrogenase (GAPDH) loading control. Statistical significance was determined using a one-way ANOVA followed by Tukey’s post hoc test for multiple comparisons (* *p* < 0.05, ** *p* < 0.01, *** *p* < 0.001, **** *p* < 0.0001).

**Figure 6:**
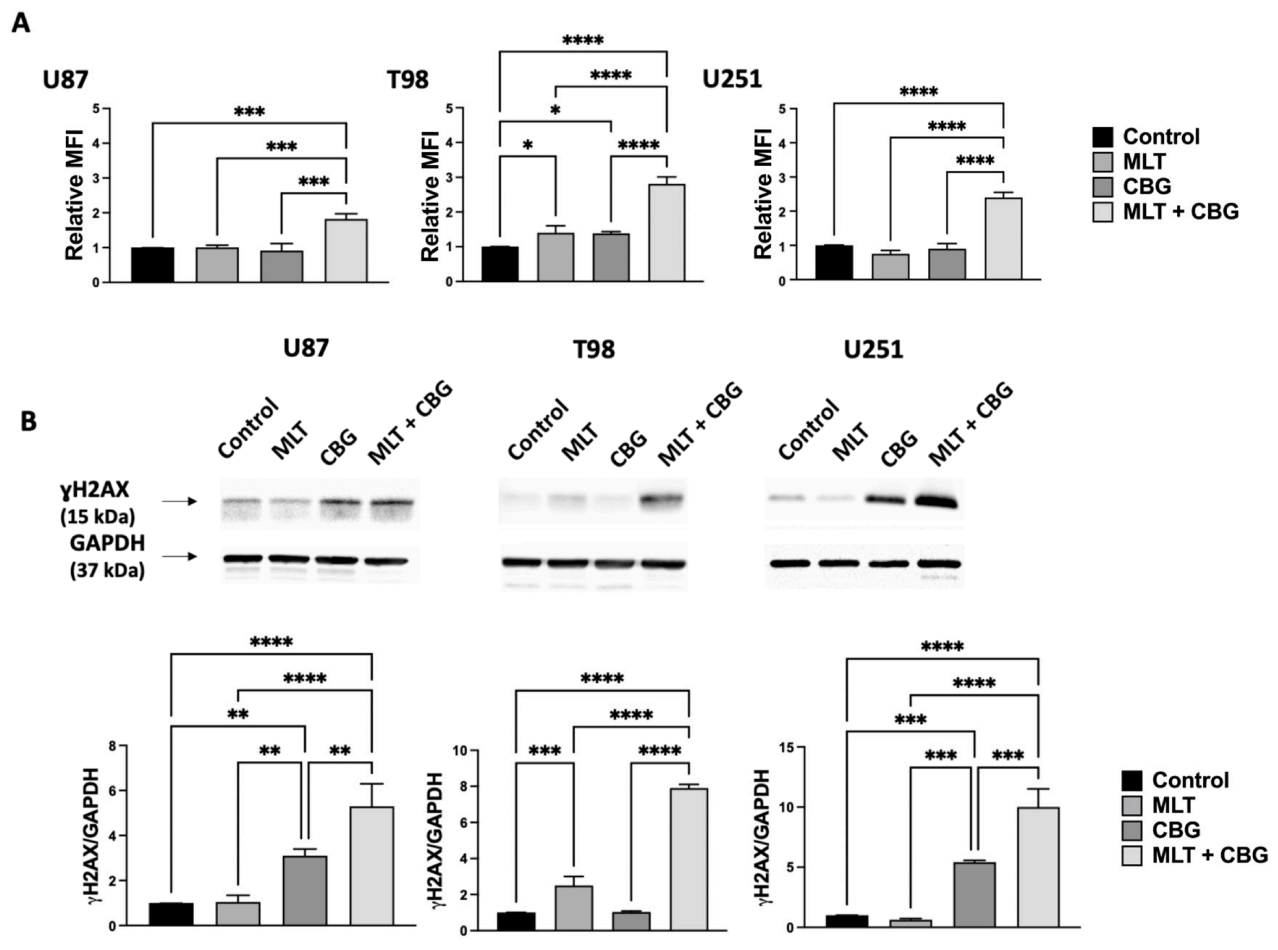
Evaluation of cell death and DNA damage in GBM cell lines following MLT and CBG, alone or in combination. GBM cell lines were treated with MLT or/and CBG for 24 h (U87: MLT 0.2 mg/mL and CBG 15 µM; T98: MLT 0.6 mg/mL and CBG 30 µM; U251: MLT 0.5 mg/mL and CBG 35 µM). (**A**) Cell death was assessed via PI staining and flow cytometry. Bar graphs represent the relative MFI, expressed as fold change relative to the untreated control for U87, T98, and U251 lines under single or combined MLT and CBN exposure. In all bar graphs, the control group was normalized to 1. Data are presented as the mean ± SD of at least three independent biological replicates. (**B**) Western blot analysis (top) and corresponding densitometric quantification (bottom) of γH2AX protein levels relative to GAPDH loading control. Statistical significance was determined using a one-way ANOVA followed by Tukey’s post hoc test for multiple comparisons (* *p* < 0.05, ** *p* < 0.01, *** *p* < 0.001, **** *p* < 0.0001).

To evaluate cell death triggered by the synergistic cannabinoid–MLT combinations, we performed flow cytometric analysis using Annexin V and Propidium Iodide (PI) staining after 24 h of exposure (Figure 5, Figure 6 and Figure S2). The 24 h time point was selected for all mechanistic and signaling analyses because it represents the optimal window to evaluate the synergistic pathways activated by the combinations prior to the onset of massive, non-specific late-stage cell death. Specifically, while Annexin V-staining was negative, co-treatment with CBN and MLT induced a substantial shift in PI fluorescence intensity across all three GBM lines (Figure 5A). Quantification of the Mean Fluorescence Intensity (MFI, expressed as fold change relative to the untreated control) confirmed a statistically significant increase in PI uptake. In U87 cells, the combination (MLT 0.3 mg/mL + CBN 25 µM) led to an approximate 3-fold increase in MFI (*p* < 0.0001), significantly bettering both single-agent treatments. A similar synergistic trend was observed in T98 cells (MLT 0.7 mg/mL + CBN 25 µM) and U251 cells (MLT 0.4 mg/mL + CBN 20 µM), where the combined groups reached a 2-fold and 1.8-fold increase in MFI, respectively (*p* < 0.001).

Paralleling these findings, the co-administration of CBG and MLT (Figure 6A) maximized PI internalization, leading to a drastic increase in MFI that reached approximately 3-fold in U87, 2-fold in T98, and 2.5-fold in U251 cells compared to their respective single-agent or control counterparts (*p* < 0.0001).

To further define the intracellular pathways involved, we evaluated key molecular markers via Western blot analysis (Figure 5B and Figure 6B). We observed a considerable upregulation of expression of the phosphorylated form of the histone H2AX (γH2AX) specifically in the groups treated with the CBN-MLT and CBG-MLT combinations. The rationale for testing this specific marker relies on its role as a well-established indicator of genomic damage. This accumulation of γH2AX demonstrates that the combined treatments induce extensive double-strand DNA breaks, which correlate with the cytotoxicity observed. To corroborate the negative apoptotic findings obtained from the flow cytometric Annexin V analysis, we also evaluated the cleavage status of Poly (ADP-ribose) Polymerase 1 (PARP-1). Western blot results showed an absence of cleaved PARP-1 under all conditions (Figure S3). Taken together, the rapid loss of membrane integrity documented by PI influx, combined with DNA damage (γH2AX-increase) and the lack of apoptotic markers, demonstrates that the cannabinoid–MLT combinations drive GBM cells toward a non-apoptotic, necrotic cell death pathway.

### 2.3. Evaluation of Oxidative Stress and Mitochondrial Homeostasis Disruption Under Combined Treatments

**Figure 7:**
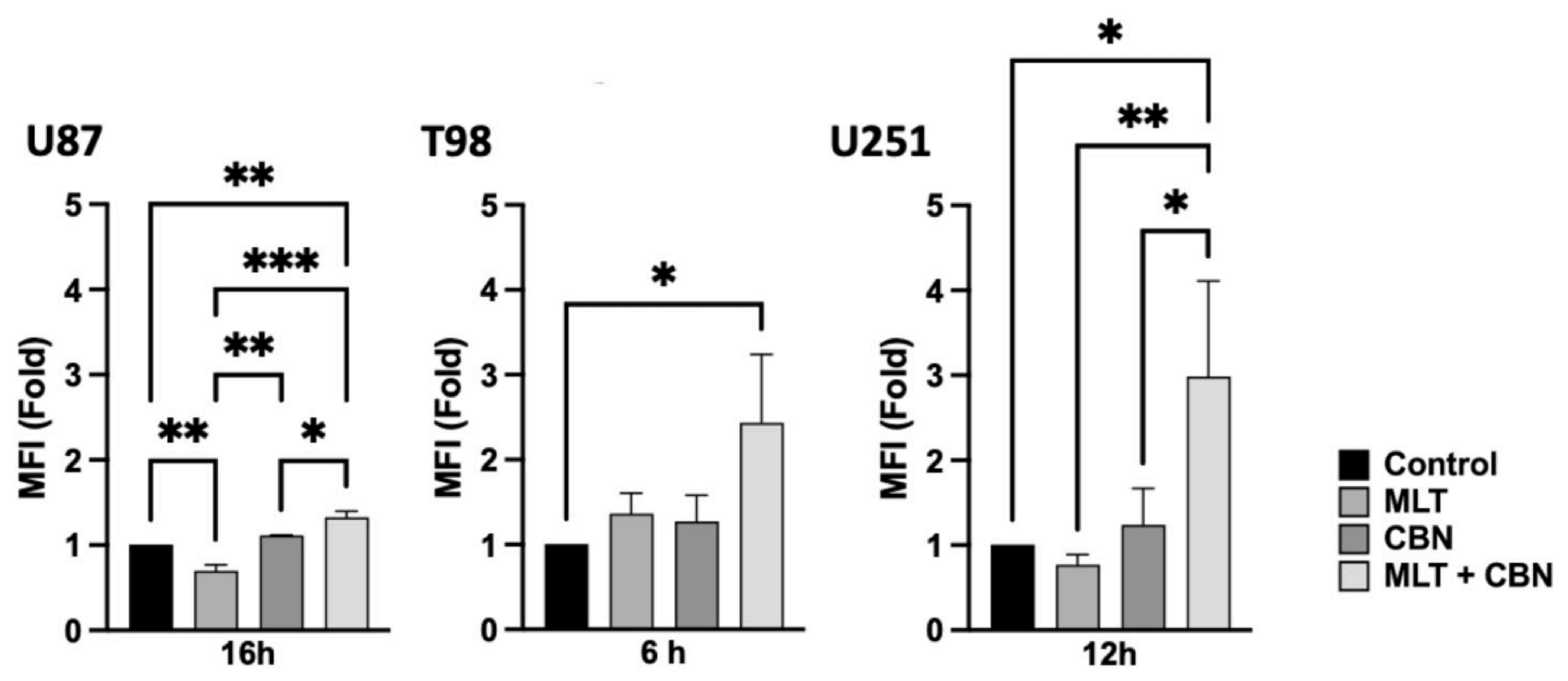
Flow cytometric analysis of intracellular ROS production in GBM cell lines under time-dependent single or combined CBN and MLT treatments (U87: MLT 0.3 mg/mL and CBN 25 µM. T98: MLT 0.7 mg/mL and CBN 25 µM. U251: MLT 0.4 mg/mL and CBN 20 µM). Quantification of DCFDA MFI, expressed as fold change relative to the untreated control, to assess intracellular ROS levels in U87 (16 h), T98 (6 h), and U251 (12 h) lines under single or combined MLT and CBN exposure. In all bar graphs, the untreated control group was normalized to 1. Data are presented as the mean ± SD of at least three independent biological replicates. Statistical significance was determined using a one-way ANOVA followed by Tukey’s post hoc test for multiple comparisons (* *p* < 0.05, ** *p* < 0.01, *** *p* < 0.001).

To investigate the mechanisms underlying the observed effects, we evaluated the induction of intracellular oxidative stress by quantifying reactive oxygen species (ROS) production via 20,70-dichlorofluorescein (DCFDA) staining and flow cytometry. The three GBM cell lines were analyzed after treatment with MLT alone, cannabinoids alone, or their respective combinations, revealing a time- and dose-dependent response. Regarding the interaction between MLT and CBN (Figure 7), the results highlighted differences among the lines. In U87 cells after 16 h of exposure, single treatment with MLT (0.3 mg/mL) significantly decreased basal ROS levels compared to the control, confirming its well-known baseline antioxidant properties. However, the introduction of 25 µM CBN profoundly altered the baseline antioxidant effect of MLT; their co-administration induced a robust and highly significant increase in ROS production, which was significantly higher than both the control and either single treatment.

A similar pro-oxidant effect was observed in T98 cells at 6 h and U251 cells at 12 h. In T98 cells, while individual treatments with MLT (0.7 mg/mL) or CBN (25 µM) failed to alter ROS production, their combination led to a statistically significant elevation compared to the control. Similarly, in U251 cells, single treatments (MLT, 0.4 mg/mL, or CBN, 20 µM) showed only a slight upward trend, but the combined regimen triggered a significant accumulation of intracellular ROS, markedly exceeding the levels observed with single MLT treatment. The different exposure times for ROS evaluation were chosen based on preliminary time-course experiments, representing the earliest time points at which significant ROS alterations were detected according to the specific kinetic and metabolic profile of each cell line.

**Figure 8:**
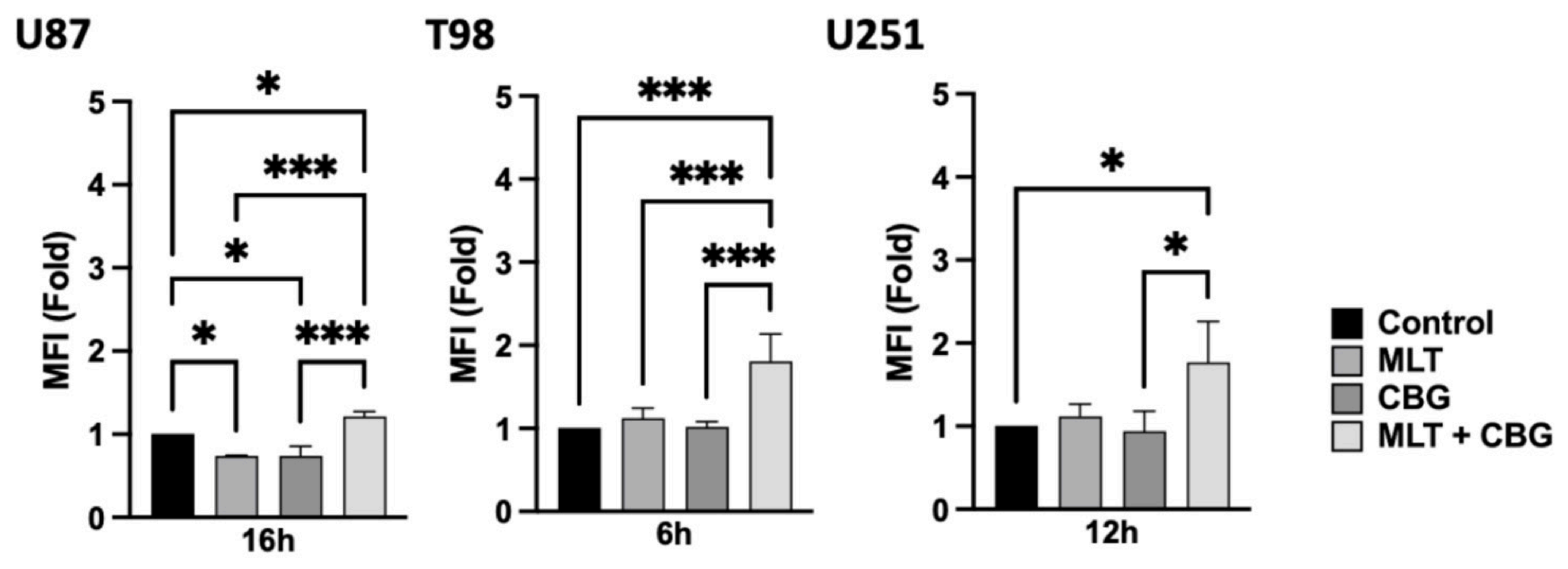
Flow cytometric analysis of intracellular ROS production in GBM cell lines under time-dependent single or combined CBG and MLT treatments (U87: MLT 0.3 mg/mL and CBG 15 µM. T98: MLT 0.6 mg/mL and CBG 30 µM. U251: MLT 0.5 mg/mL and CBG 35 µM). Quantification of DCFDA MFI, expressed as fold change relative to the untreated control, to assess intracellular ROS levels in U87 (16 h), T98 (6 h), and U251 (12 h) lines under single or combined MLT and CBG exposure. In all bar graphs, the untreated control group was normalized to 1. Data are presented as the mean ± SD of at least three independent biological replicates. Statistical significance was determined using a one-way ANOVA followed by Tukey’s post hoc test for multiple comparisons (* *p* < 0.05, *** *p* < 0.001).

An almost identical profile of oxidative stress activation emerged when evaluating the combination of MLT and CBG (Figure 8). In U87 cells, single MLT (0.2 mg/mL) treatment again exerted a scavenger-like reduction in baseline ROS, but the co-administration with 15 µM CBG switched this response toward a potent pro-oxidant action, significantly driving up ROS levels compared to all other experimental groups. In T98 cells, the combination of MLT (0.6 mg/mL) and 30 µM CBG promoted a statistically significant upregulation of oxidative stress, clearly better than both single compounds. Lastly, in the U251 line, the combination of MLT (0.5 mg/mL) and 35 µM CBG induced a significant shift in ROS levels relative to the control. Taken together, these findings demonstrate that despite the initial antioxidant behavior of MLT seen in specific settings like the U87 line, its combination with either CBN or CBG shifts the cellular environment toward a strong pro-oxidant state across all tested GBM cell lines, suggesting that these combinations trigger specific intracellular oxidative stress pathways.

**Figure 9:**
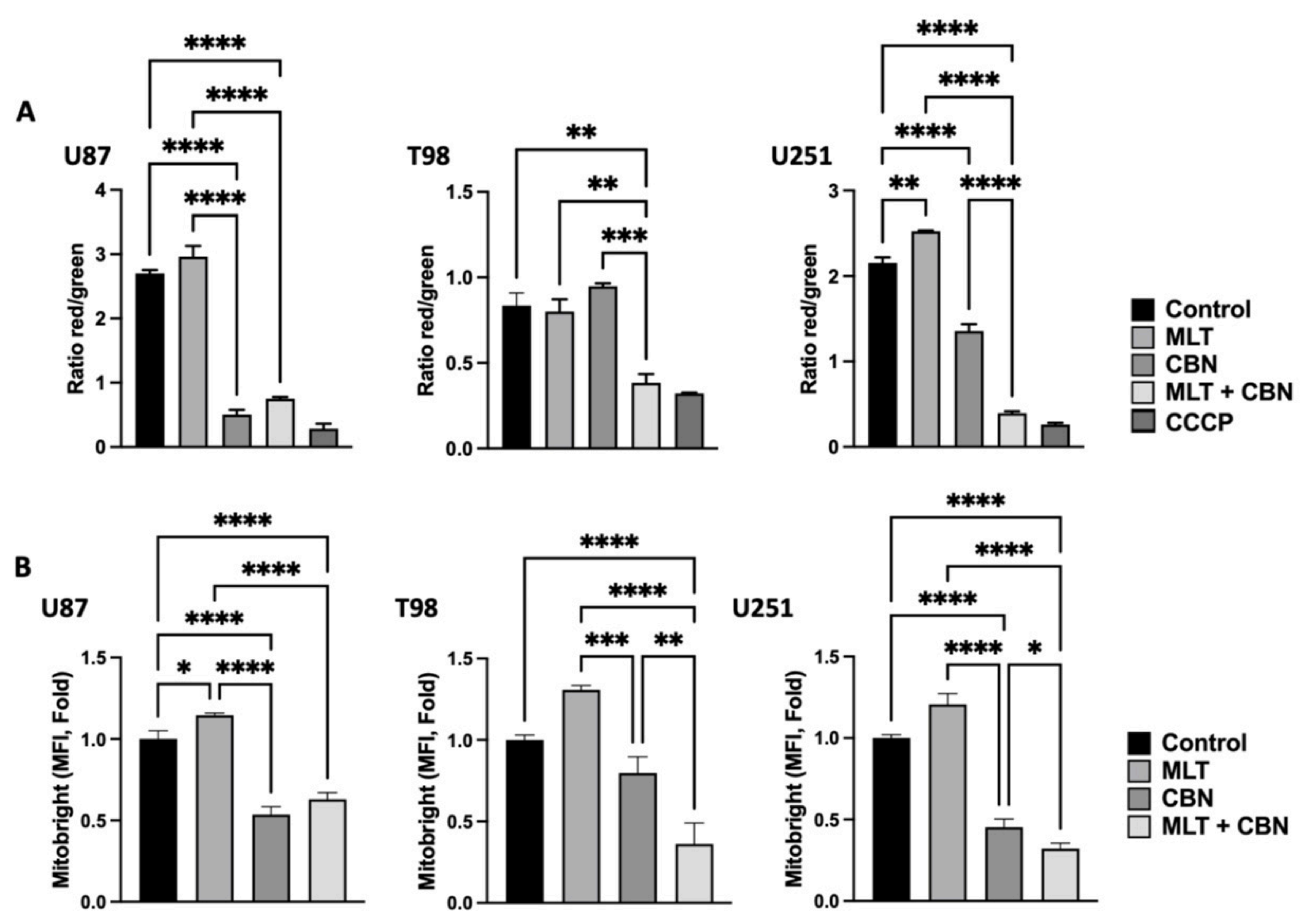
Effects of CBN and MLT treatments on mitochondrial status in GBM cells. (**A**) Flow cytometric analysis of mitochondrial membrane potential (Δψ_m_) expressed as the red (FL2)/green (FL1) fluorescence intensity ratio of JC-1 staining in U87, T98, and U251 lines after 24 h treatment with single or combined agents (U87: MLT 0.3 mg/mL and CBN 25 µM. T98: MLT 0.7 mg/mL and CBN 25 µM. U251: MLT 0.4 mg/mL and CBN 20 µM). CCCP was used as a positive control for mitochondrial depolarization. (**B**) Flow cytometric quantification of mitochondrial mass assessed via MitoBright staining (MFI, fold change) in U87, T98 and U251 lines following single or combined MLT and CBN treatments. In all bar graphs, data are presented as the mean ± SD of at least three independent biological replicates. Statistical significance was determined using a one-way ANOVA followed by Tukey’s post hoc test for multiple comparisons (* *p* < 0.05, ** *p* < 0.01, *** *p* < 0.001, **** *p* < 0.0001).

To determine whether the observed pro-oxidant effects were associated with mitochondrial dysfunction, we evaluated changes in mitochondrial membrane potential (Δψ_m_) and mitochondrial dynamics across the GBM cell lines after 24 h of treatment. Mitochondrial membrane potential was assessed by calculating the red/green fluorescence ratio of 5,5′,6,6′-tetrachloro-1,1′,3,3′-tetraehylbenzimidazolylcarbocyanineiodide (JC-1). When examining the combination of MLT and CBN (Figure 9A), a distinct line-dependent pattern of depolarization emerged. In U87 cells, single treatments with either MLT (0.3 mg/mL) or CBN (25 µM) led to an increase in the red/green ratio compared to the control, whereas their combination significantly reduced this ratio, indicating an induction of mitochondrial stress with co-administration. In T98 cells, single treatments did not induce depolarization, whereas in U251 cells, single administrations triggered it; however, the combined administration of MLT and CBN induced a highly significant collapse of Δψ_m_ in both lines (*p* < 0.001). In all experimental settings, the uncoupler carbonyl cyanide chlorophenylhydrazone protonophore (CCCP) was utilized as a positive control, confirming effective mitochondrial depolarization.

**Figure 10:**
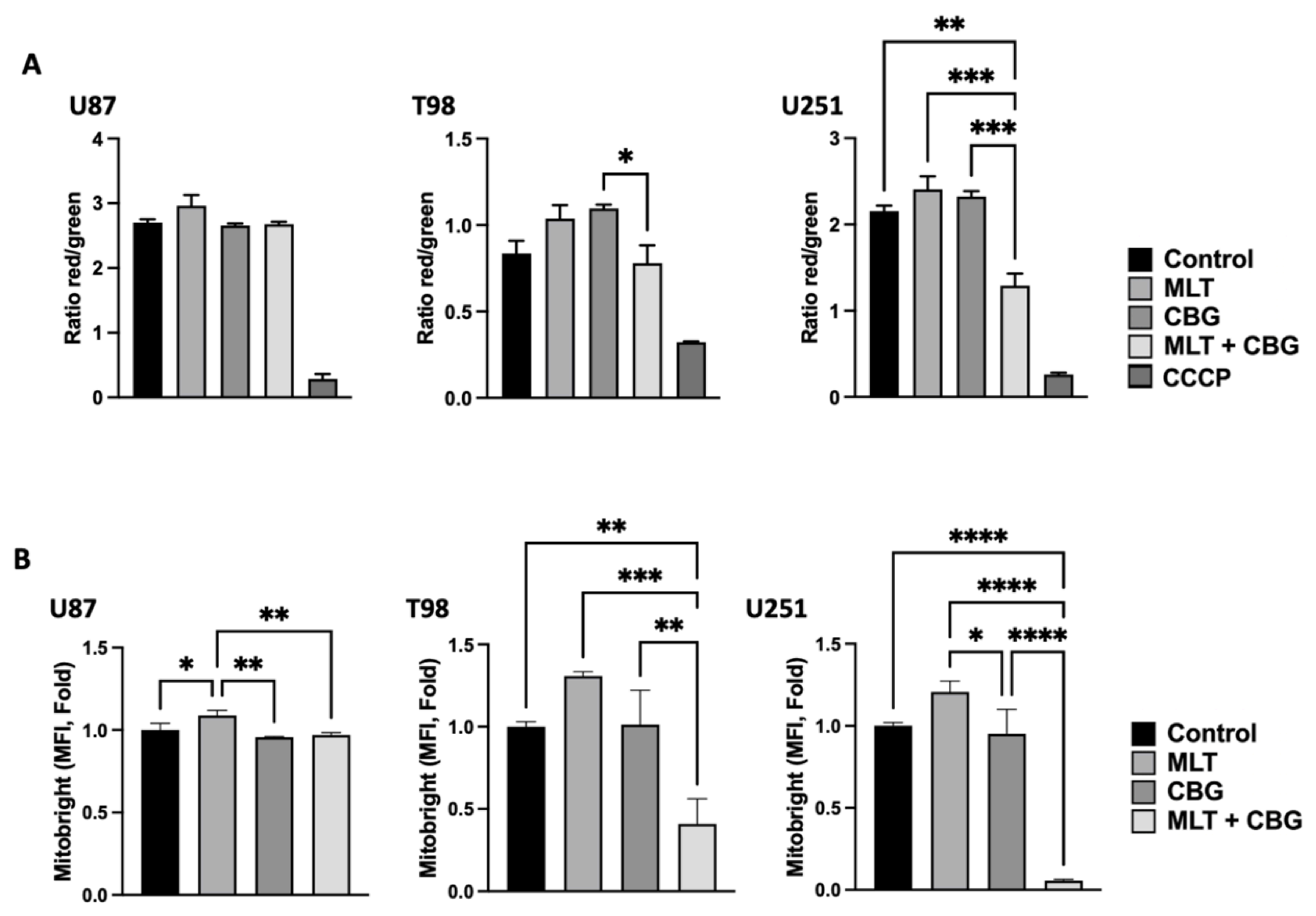
Effects of CBG and MLT treatments on mitochondrial status in GBM cells. (**A**) Flow cytometric analysis of mitochondrial membrane potential expressed as the red (FL2)/green (FL1) fluorescence intensity ratio of JC-1 staining in U87, T98, and U251 lines after 24 h treatment with single or combined agents (U87: MLT 0.3 mg/mL and CBG 15 µM. T98: MLT 0.6 mg/mL and CBG 30 µM. U251: MLT 0.5 mg/mL and CBG 35 µM). CCCP was used as a positive control for mitochondrial depolarization. (**B**) Flow cytometric quantification of mitochondrial mass assessed via MitoBright staining (MFI, fold change) in U87, T98 and U251 lines following single or combined MLT and CBG treatments. In all bar graphs, data are presented as the mean ± SD of at least three independent biological replicates. Statistical significance was determined using a one-way ANOVA followed by Tukey’s post hoc test for multiple comparisons (* *p* < 0.05, ** *p* < 0.01, *** *p* < 0.001, **** *p* < 0.0001).

A partially overlapping trend was observed when evaluating the combination of MLT and CBG (Figure 10A). While U87 cells remained resistant to depolarization under these specific conditions, T98 cells exposed to the combination showed a significant decrease in the JC-1 ratio compared to the single CBG treatment (*p* < 0.05). Notably, U251 cells exhibited a highly sensitive response to the MLT and CBG combination, resulting in a statistically significant reduction in Δψ_m_ relative to both the untreated control and the single agents (*p* < 0.001).

To further characterize these mitochondrial alterations, we monitored changes in mitochondrial mass or dynamics using MitoBright staining (Figure 9B and Figure 10B). In T98 cells, single treatments with MLT (0.7 mg/mL) caused a modest increase in MitoBright fluorescence, but CBN (25 µM) or the combination significantly suppressed this signal (Figure 9B). A similar and more pronounced effect was observed in U251 cells, where the combined treatment led to a reduction in mitochondrial staining (*p* < 0.05), suggesting an acceleration of mitochondrial degradation or a severe impairment of mitochondrial biogenesis. In U87 cells, CBN (25 µM) was able to markedly decrease the MitoBright fluorescence; however, the combination did not improve the reduction. Moreover, in U251 and T98 cells, the combination CBG and MLT strongly reduces the MitoBright signal more than single treatments (*p* < 0.001) (Figure 10B).

**Figure 11:**
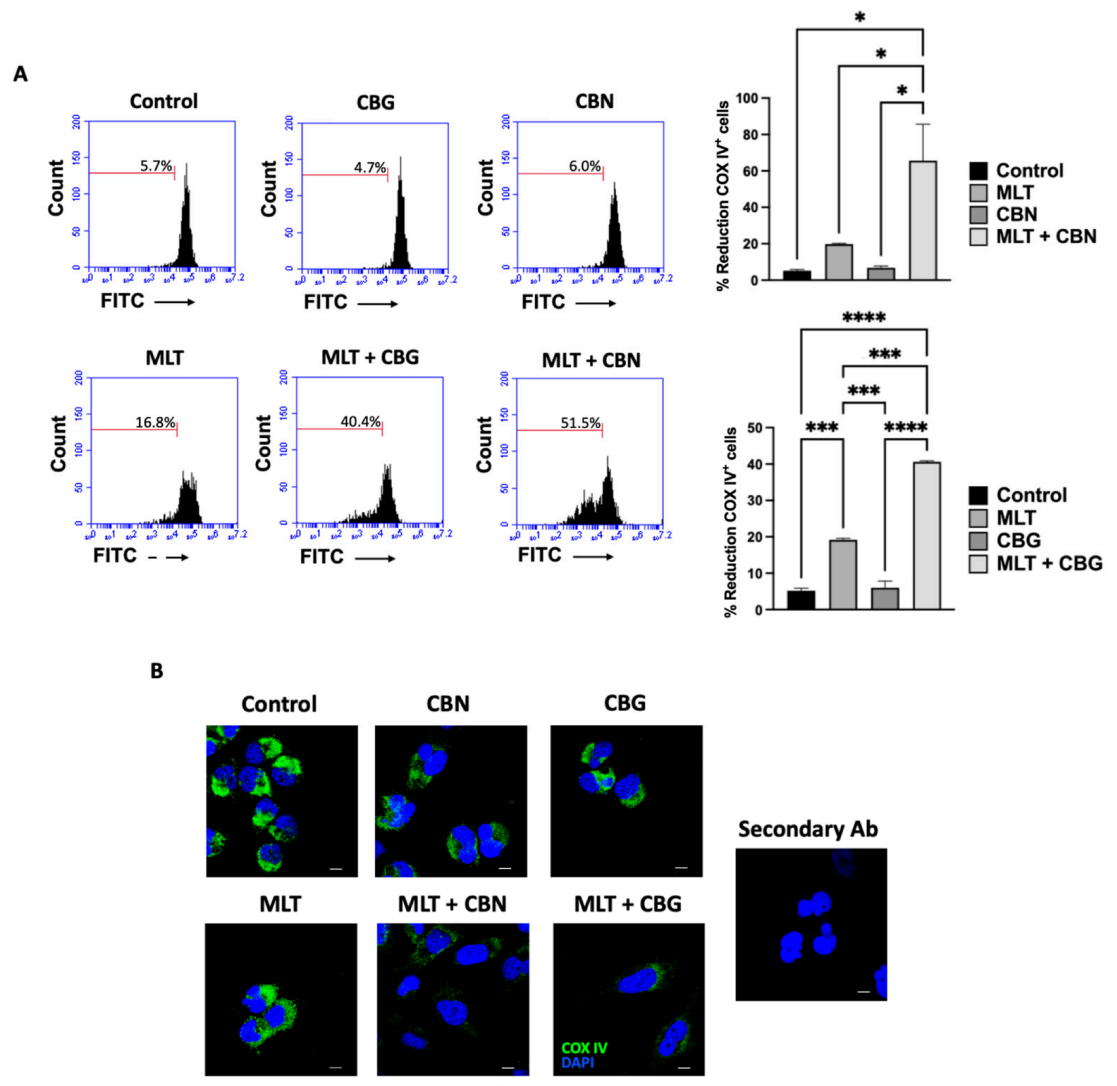
Effect of individual or combined MLT and phytocannabinoid treatments on COX IV expression. (**A**) Flow cytometric analysis of Cytochrome c Oxidase subunit IV (COX-IV) expression in T98 cell line under the respective single or combined cannabinoids or MLT treatments (MLT 0.7 mg/mL and CBN 25 µM; MLT 0.6 mg/mL and CBG 30 µM). Markers represent the percentage reduction of COX IV-positive cells, as quantified in the accompanying bar graphs. (**B**) Confocal microscopy analysis of COX-IV expression (green fluorescence), with nuclei counterstained using diamidino-2-phenylindole (DAPI, blue fluorescence), and localization in T98 cells (magnification 100×; scale bar = 10 µm). In all bar graphs, data are presented as the mean ± SD of at least three independent biological replicates. Statistical significance was determined using a one-way ANOVA followed by Tukey’s post hoc test for multiple comparisons (* *p* < 0.05, *** *p* < 0.001, **** *p* < 0.0001).

Finally, we investigated whether this mitochondrial impairment correlated with the expression of Cytochrome c Oxidase (Figure 11 and Figure S4). Flow cytometric analysis of COX subunit IV (COX-IV) expression (Figure 11A) revealed a significant downregulation following the combined treatments with both CBN and CBG in T98 and U251 cells and with CBN in U87, confirming the previous results. To visually validate these findings, confocal microscopy was performed to confirm COX-IV expression and localization in T98 cells (Figure 11B), which qualitatively confirmed a distinct reorganization and reduction in the mitochondrial metabolic enzyme under combined treatment conditions. Taken together, the T98 and U251 lines proved highly susceptible to mitochondrial impairment, leading to severe bioenergetic collapse. Conversely, U87 cells demonstrated a marked resistance to combination-induced mitochondrial dysfunction after treatment with CBG, showing no significant alterations in membrane potential, organelle mass, or COX-IV expression after 24 h of treatment.

**Figure 12:**
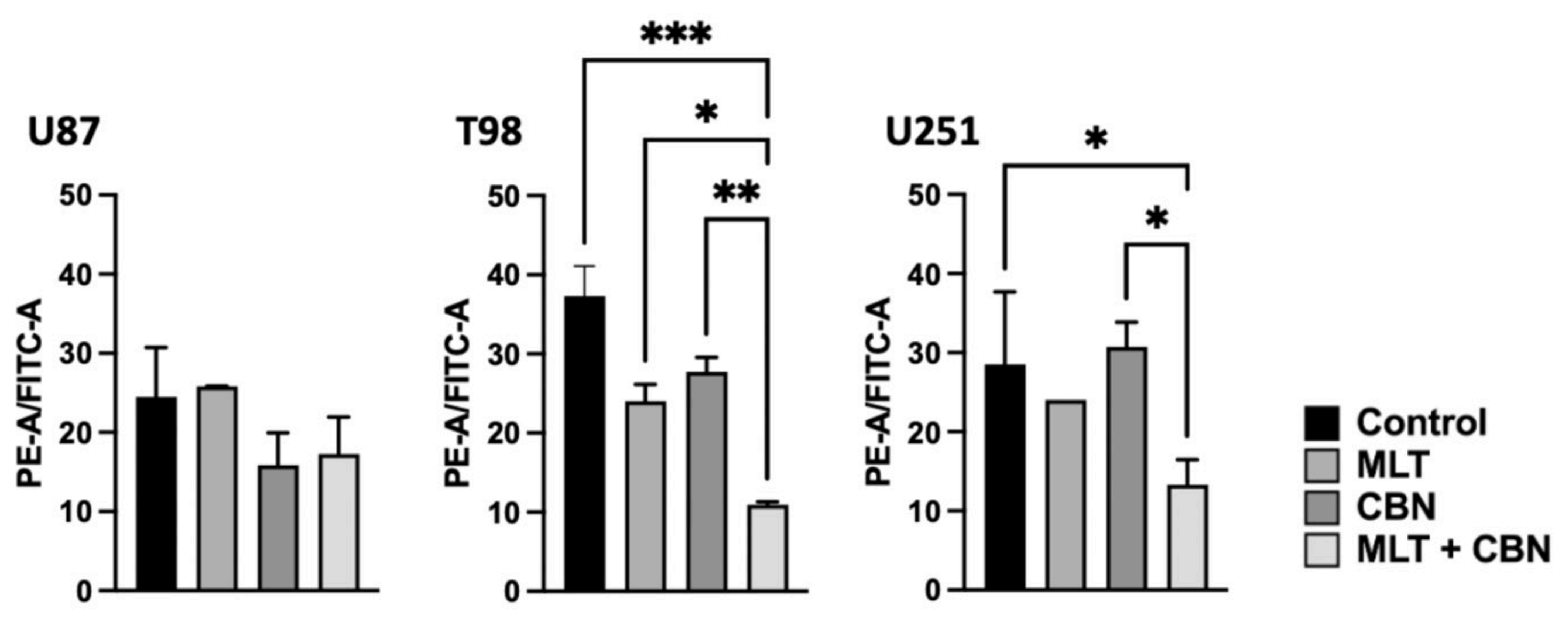
Flow cytometric analysis of lipid peroxidation in GBM cell lines under single or combined CBN and MLT treatments. Evaluation of lipid peroxidation expressed as the red/green fluorescence ratio of BODIPY staining in U87, T98, and U251 lines after 24 h treatment with single or combined MLT and CBN (U87: MLT 0.3 mg/mL and CBN 25 µM. T98: MLT 0.7 mg/mL and CBN 25 µM. U251: MLT 0.4 mg/mL and CBN 20 µM). In all bar graphs, data are presented as the mean ± SD of at least three independent biological replicates. Statistical significance was determined using a one-way ANOVA followed by Tukey’s post hoc test for multiple comparisons (* *p* < 0.05, ** *p* < 0.01, *** *p* < 0.001).

**Figure 13:**
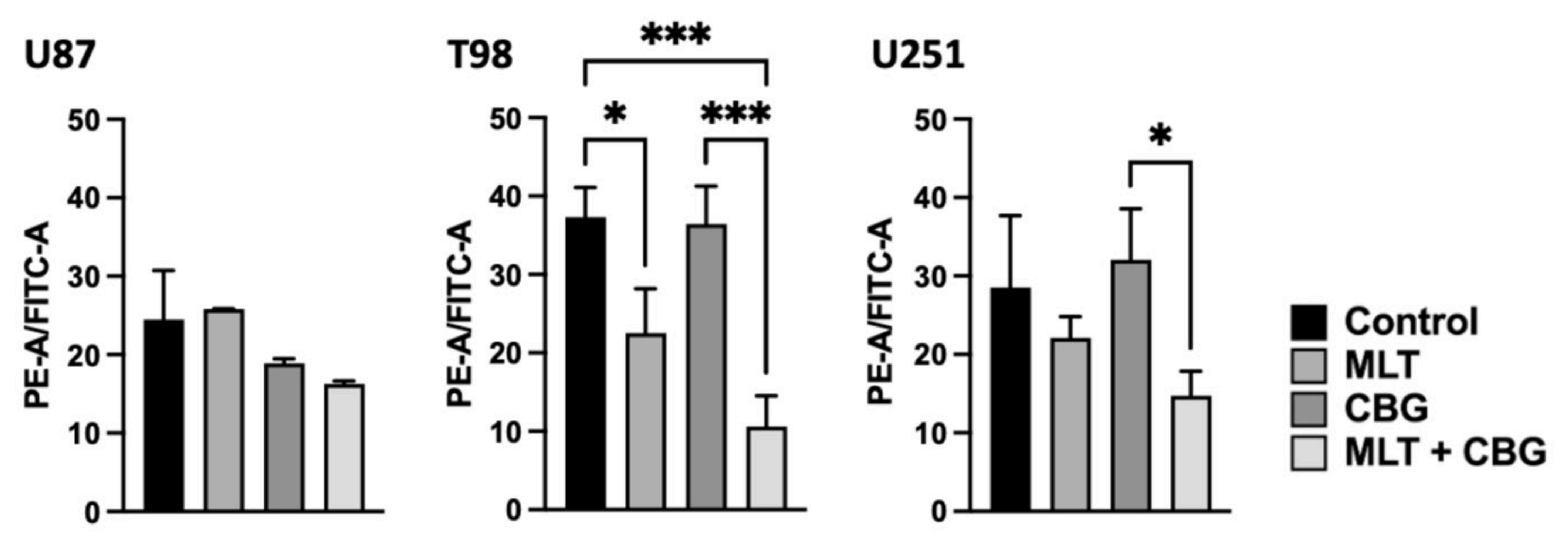
Flow cytometric analysis of lipid peroxidation in GBM cell lines under single or combined CBG and MLT treatments. Evaluation of lipid peroxidation expressed as the red/green fluorescence ratio of staining in U87, T98, and U251 lines after 24 h treatment with single or combined MLT and CBG (U87: MLT 0.3 mg/mL and CBG 15 µM. T98: MLT 0.6 mg/mL and CBG 30 µM. U251: MLT 0.5 mg/mL and CBG 35 µM). In all bar graphs, data are presented as the mean ± SD of at least three independent biological replicates. Statistical significance was determined using a one-way ANOVA followed by Tukey’s post hoc test for multiple comparisons (* *p* < 0.05, *** *p* < 0.001).

To evaluate whether the intracellular ROS accumulation led to structural membrane damage, we investigated lipid peroxidation levels across the three GBM cell lines after 24 h of treatment using the fluorescent dye BODIPY (Figure 12 and Figure 13). The analysis revealed a strong, line-dependent susceptibility to lipid peroxidation that closely mirrored the mitochondrial damage profile. Consistent with their general resistance, U87 cells did not display any significant alterations in the BODIPY red/green ratio under single or combined treatments with either CBN (25 µM, Figure 12) or CBG (15 µM, Figure 13). Conversely, both T98 and U251 cell lines were highly sensitive to treatment-induced lipid oxidation. When exposed to the combination of MLT (0.7 mg/mL) and CBN (25 µM), T98 cells exhibited a significant decrease in the red/green ratio compared to the untreated control (*p* < 0.001) and to the single CBN treatment. A similar significant drop was observed in U251 cells treated with the MLT (0.4 mg/mL) and CBN (20 µM) in combination compared to both the control and single cannabinoid exposure (*p* < 0.05) (Figure 12).

When evaluating the MLT-CBG combination, co-administration with MLT triggered lipid peroxidation in T98 (*p* < 0.001) and U251 cells relative to their respective single cannabinoid treatments (Figure 13).

Since lipid peroxidation is a key hallmark of ferroptosis, an iron-dependent form of regulated cell death [35], we investigated whether this pathway was driving the observed cytotoxicity in T98 and U251 cells. To test this hypothesis, cells were pre-treated with the specific ferroptosis inhibitor Liproxstatin-1 prior to exposure to the single or combined compounds. Interestingly, the pharmacological blockade of ferroptosis failed to rescue or attenuate the cytotoxic effects induced by either CBN, CBG, or their combinations with MLT (Figure S5).

In conclusion, while in U87 cells the cytotoxic effect of the combinations is not associated with lipotoxic injury, the combination of MLT with cannabinoids triggers substantial lipid membrane oxidation in T98 and U251 lines; however, this lipid peroxidation does not initiate a ferroptotic cascade, further confirming that cell death proceeds through a necrotic pathway.

### 2.4. The Addition of Cannabinoid–MLT Combinations Enhances TMZ Effects on Cell Viability

**Figure 14:**
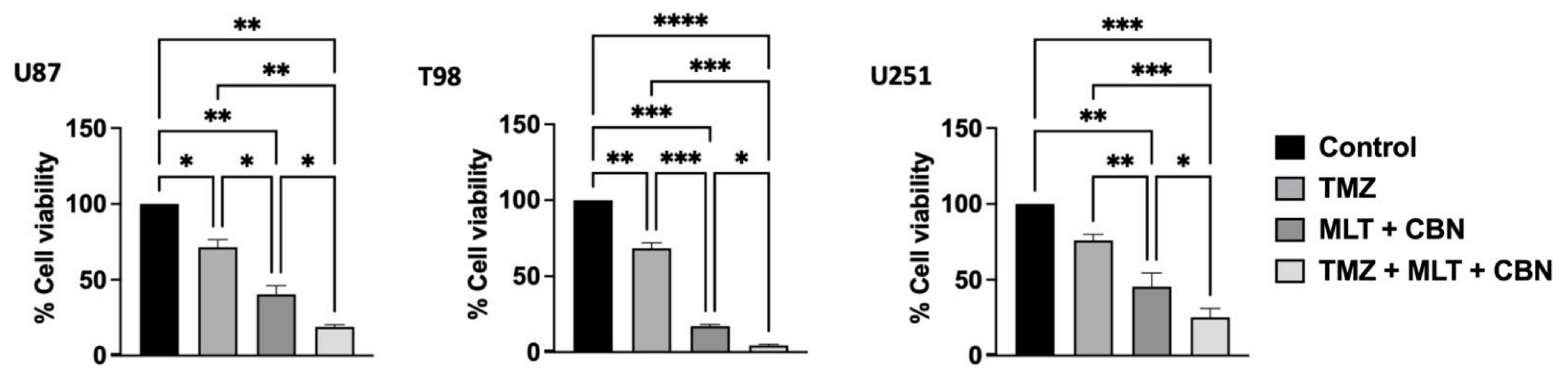
Combined treatment of MLT and CBN with TMZ enhances cell viability reduction in GBM cells. Cell viability was assessed by MTT assay after 72 h of treatment to evaluate the cytotoxic effects of TMZ (600 µM) alone, MLT combined with CBN (U87: MLT 0.3 mg/mL and CBN 25 µM. T98: MLT 0.7 mg/mL and CBN 25 µM. U251: MLT 0.4 mg/mL and CBN 20 µM), or the triple combination (TMZ + MLT + CBN). Vehicle-treated cells were used as a control group (100% viability). Data are expressed as the mean ± SD of 3 independent experiments. Statistical significance was determined using one-way ANOVA followed by Tukey’s post hoc test (* *p* < 0.05, ** *p* < 0.01, *** *p* < 0.001, **** *p* < 0.0001).

**Figure 15:**
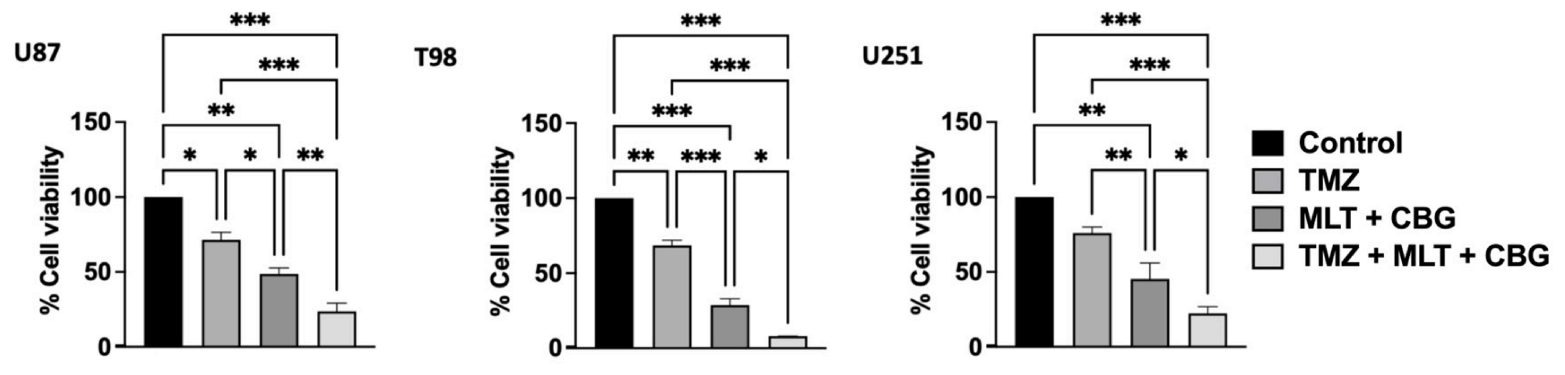
Combined treatment of MLT and CBG with TMZ enhances cell viability reduction in GBM cells. Cell viability was assessed by MTT assay after 72 h of treatment to evaluate the cytotoxic effects of TMZ (600 µM) alone, MLT combined with CBG (U87: MLT 0.3 mg/mL and CBG 15 µM. T98: MLT 0.6 mg/mL and CBG 30 µM. U251: MLT 0.5 mg/mL and CBG 35 µM), or the triple combination (TMZ + MLT + CBG). Vehicle-treated cells were used as a control group (100% viability). Data are expressed as the mean ± SD of 3 independent experiments. Statistical significance was determined using one-way ANOVA followed by Tukey’s post hoc test (* *p* < 0.05, ** *p* < 0.01, *** *p* < 0.001).

To evaluate whether the co-administration of cannabinoid–MLT combinations could boost the efficacy of standard chemotherapy, we tested the effects of TMZ in combination with either MLT + CBN or MLT + CBG across GBM cell lines (Figure 14 and Figure 15). As shown, treatment with TMZ alone led to a moderate reduction in cell viability across the tested lines. However, the combination of MLT and CBN induced a significantly stronger inhibitory effect compared to TMZ alone (*p* < 0.05) (Figure 14). Crucially, the deployment of the triple combination (TMZ + MLT + CBN) led to the lowest percentage of cell viability, significantly outperforming both TMZ alone (*p* < 0.01) and the MLT + CBN treatment (*p* < 0.05). These results are consistently observed in the U87, T98 and U251 cell lines, demonstrating that the chemotherapy agent works cooperatively with the MLT + CBN to maximize the inhibitory effects.

A highly comparable pattern was observed when testing MLT + GBG (Figure 15). While the MLT + CBG combination already exerted robust cytotoxicity (*p* < 0.01 vs. control, *p* < 0.05 vs. TMZ), its co-administration with TMZ (TMZ + MLT + CBG) triggered a further significant drop in cell viability in all tested GBM models (*p* < 0.01 vs. TMZ alone; *p* < 0.05 vs. MLT + CBG).

Taken together, these findings indicate that MLT–cannabinoid combinations significantly enhance the cytotoxic profile of TMZ, leading to a more pronounced reduction in GBM cell viability when administered as a triple combination.

## 3. Discussion

A wide range of natural and endogenous compounds with well-established biological activities have demonstrated anti-tumor effects against GBM in both in vitro and in vivo preclinical models [36]. Combination therapies employing these bioactive molecules have gained increasing attention because they simultaneously target multiple oncogenic pathways while exhibiting relatively low toxicity toward normal tissues. To address this critical need for novel multi-targeted strategies against the highly challenging landscape of GBM, this study investigates the innovative anti-tumor potential of combining MLT with minor phytocannabinoids. Our principal findings demonstrate that both MLT-CBN and MLT-CBG combinations exert synergistic cytotoxic effects across three genetically distinct GBM cell lines, particularly when administered at intermediate-to-high dose ranges. Crucially, this cytotoxic efficacy triggers a collapse in cell viability in both p53 wild-type (U87) and p53-mutated/deficient (T98 and U251) backgrounds. Given that p53 mutations commonly drive resistance to conventional alkylating agents like TMZ, the ability to induce cell death regardless of p53 status underscores the translational relevance of these combinations in overcoming standard chemoresistance [37]. Supporting this, our results show that combining MLT with either CBN or CBG significantly enhances the basal cytotoxic profile of TMZ, leading to a much more pronounced reduction in GBM cell viability across all the tested cell lines. Collectively, these findings highlight the potential of these novel combinations to overcome the therapeutic limitations that currently characterize GBM treatments.

Crucially, an essential requirement for any anti-GBM strategy is cell-type selectivity, ensuring that the therapeutic agents target malignant tissue while sparing the surrounding healthy brain parenchyma. To address this pivotal translational aspect and validate the safety profile of our approach, we tested the combinations on human astrocytes. The MLT-CBN/CBG combinations exhibited variable selectivity, resulting in an SI ranging from 1.00 up to 2.04. In vitro, an SI equal to 1.0 indicates equivalent cytotoxicity between tumor and non-malignant cells, whereas an SI greater than 1.0 indicates a favorable profile where tumor-directed efficacy outweighs toxicity toward healthy cells. Specifically, an SI value between 1.1 and 2.0 represents a narrow yet positive therapeutic window, demonstrating that the combinations can achieve up to a twofold higher selectivity for specific GBM lines. Although some combinations show overlapping toxicity profiles (SI = 1.00), in the context of GBM, where conventional alkylating agents such as TMZ are limited by severe, dose-dependent systemic toxicity, even a narrow or cell-line-specific increase in tumor selectivity carries important clinical implications. Because both phytocannabinoids and MLT are well-tolerated in vivo, and have shown high safety profiles even at elevated therapeutic doses in clinical and preclinical models [24,30,38,39,40], this net selectivity points to a manageable therapeutic window capable of targeting the tumor phenotype while minimizing collateral damage to surrounding tissues, thereby highlighting a favorable safety profile essential for future translational studies.

MLT exerts pleiotropic inhibitory effects on cancer cells by regulating mitochondrial function, apoptosis, autophagy, cellular metabolism, and PI3K/AKT/mTOR and NF-κB signaling while acting as a chemosensitizing agent [41]. In parallel, phytocannabinoids induce cytotoxicity via receptor-dependent and -independent mechanisms, triggering oxidative stress, mitochondrial dysfunction, and endoplasmic reticulum stress [42]. Consequently, the enhanced cytotoxicity of the combination likely results from a simultaneous impairment of mitochondrial homeostasis, redox balance, and cell survival pathways. This synergistic interaction supports the concept that MLT can potentiate the activity of natural phytochemicals through complementary, non-overlapping molecular mechanisms [41]. Mechanistically, our data demonstrate that these combinations do not rely on the apoptotic pathway but lead to GBM cell death characterized by a rapid loss of cell membrane integrity. In the context of GBM therapy, driving cells toward a p53-independent necrotic pathway represents a highly advantageous clinical trait. Mutations in the TP53 gene are among the most frequent alterations in GBM, driving aggressive tumor progression and conferring profound resistance to conventional pro-apoptotic chemotherapies, which rely on functional p53 signaling to trigger cell death [37]. By triggering necrotic cell death irrespective of the tumor’s genetic background, the MLT-CBN and MLT-CBG combinations offer a powerful strategy to overcome intrinsic drug resistance. This mechanism reinforces their potential as promising multi-target candidates for future preclinical validation, particularly in tumors where conventional apoptotic signaling is severely impaired.

To dissect the upstream triggers of this necrotic death, we investigated the interplay between oxidative stress, mitochondrial homeostasis, and membrane integrity. Our results unraveled a line-dependent response. In the T98 and U251 cells, the co-administration of MLT with CBN or CBG induced an early, massive accumulation of ROS. This overwhelming oxidative burst directly targeted the mitochondria, prompting a highly significant collapse of the mitochondrial membrane potential and a depletion of mitochondrial mass. These events culminated in extensive lipotoxicity, as documented by a severe drop in the BODIPY red/green ratio, indicating widespread lipid peroxidation of structural membranes. Within these responsive cells, CBN-containing regimens consistently overtake CBG-based treatments, identifying CBN as a significantly more potent driver of mitochondrial structural and functional dismantling. In contrast, the U87 cell line exhibited a distinct response depending on the minor cannabinoid. Both combinations induced cytotoxicity and necrotic cell death; however, CBN and CBG elicited different death-associated signaling after 24 h of treatment. CBN induced ROS production and mitochondrial depolarization, consistent with the responses observed in T98 and U251 cells. In contrast, CBG did not induce oxidative stress, mitochondrial depolarization, mitochondrial mass loss, or early lipid peroxidation, suggesting activation of distinct cell death signaling pathways. The divergence between cell lines’ behavior may be attributed to the higher basal antioxidant capacity of U87 cells, which has been previously reported to confer greater resistance to mild oxidative insults [43], while the distinct redox responses elicited by CBN and CBG in U87 cells may reflect differences in both their molecular targets or receptor engagement and the magnitude of oxidative stress they induce. Further investigation of mitochondrial respiratory function, antioxidant defense systems, and stress-responsive signaling pathways will be required to elucidate the molecular mechanisms underlying these differential responses in the U87 glioma cell line.

In summary, this study provides a strong foundation for combining minor cannabinoids and MLT to exploit their synergistic cytotoxic effects against GBM. These findings bridge the gap between empirical case reports and rigorous molecular oncology, offering a promising approach to complement conventional GBM strategies.

Despite the promising synergistic effects observed, several limitations of this study must be acknowledged to contextualize its clinical translation. First, these results were obtained strictly in cell culture models, which cannot fully replicate the complex, cellularly heterogeneous, and immunosuppressive microenvironment of GBM in vivo. Second, ensuring adequate brain bioavailability represents a major pharmacological hurdle. While CBG and CBN possess favorable properties for passive diffusion across the BBB, their high lipophilicity classifies them as Biopharmaceutics Classification System (BCS) class II drugs, characterized by poor aqueous solubility, rapid hepatic distribution, and high susceptibility to active efflux mechanisms [44,45]. Similarly, although MLT readily crosses the BBB [46], its therapeutic efficacy via conventional routes is severely hindered by a short plasma half-life and extensive first-pass metabolism. Additionally, for future pharmaceutical scale-up, chemical synthesis will represent a more suitable option than plant extraction to guarantee standardized purity and scalability [47]. Finally, the complex and fragmented regulatory framework surrounding minor cannabinoids across different jurisdictions poses non-trivial challenges for standardization and clinical trial designs.

To address these gaps, the next essential research steps will require the development of novel nanotechnology-based delivery systems to overcome pharmacokinetic hurdles and optimize brain delivery [48]. Also, alternative administrative routes, particularly intranasal delivery, could exploit the olfactory pathway to achieve direct nose-to-brain drug transport. Furthermore, future animal studies will be mandatory to thoroughly evaluate the pharmacokinetics, biodistribution, and BBB penetration of these combinations, as well as their therapeutic efficacy and safety profile in a living organism. However, it is worth noting that the translational potential of this specific cannabinoid–MLT combination is already supported by encouraging clinical case reports showing positive outcomes in human patients [25,26,27,28,29]. Thus, while formal preclinical validation is ongoing, these real-world data strongly reinforce the clinical relevance of our mechanistic findings.

## 4. Materials and Methods

### 4.1. Cell Lines

Human GBM cell lines U87, T98, and U251 (European Collection of Cell Cultures, Salisbury, UK) were maintained in Eagle’s Minimum Essential Medium (EMEM; S.I.A.L., Milan, Italy) enriched with 10% (*v*/*v*) heat-inactivated fetal bovine serum (FBS, S.I.A.L.), penicillin (100 IU/mL, S.I.A.L.), streptomycin (100 μg/mL, S.I.A.L.), 2 mmol/L L-glutamine (S.I.A.L.), 10% (*v*/*v*) nonessential amino acids (S.I.A.L.), and 10% (*v*/*v*) sodium pyruvate (S.I.A.L.). The Immortalized Human Adult Brain Astrocytes (AST-1) (NHA, Applied Biological Materials Inc., Richmond, BC, Canada) were cultured in PriGrow IV medium (TM004, Applied Biological Materials Inc.), 10% FBS + 10 ng/mL human Epidermal Growth Factor (EGF, Applied Biological Materials Inc), 10 ng/mL human Hepatocyte Growth Factor (HGF, Applied Biological Materials Inc.), 10 ng/mL human Insulin-like Growth Factor (IGF-1, Applied Biological Materials Inc.), penicillin (100 IU/mL), and streptomycin (100 μg/mL). All cultures were incubated at 37 °C under humidified conditions with 5% CO_2_. All cell cultures were routinely tested for mycoplasma contamination using the PCR Mycoplasma Detection & Elimination kit (Applied Biological Materials Inc.) and remained strictly negative throughout the study.

### 4.2. Compounds

MLT (Cayman Chemical, Ellsworth, MI, USA) was prepared fresh by dissolving in 70% ethanol at 40 mg/mL. Pharmaceutical-grade CBG and CBN crystals were purchased from Cayman Chemical and solubilized in 70% ethanol (S.I.A.L.) at 50 mM. TMZ was dissolved in DMSO (S.I.A.L.) to obtain a 50 mM stock solution (Sigma-Aldrich, Milan, Italy). Liproxstatin-1 was dissolved in DMSO to obtain a 29.3384 mM stock solution (Sigma-Aldrich). Aliquots were prepared and stored at −20 °C; each compound aliquot was for single use only to avoid freeze–thaw degradation. For all experiments, appropriate vehicle controls (containing the maximum final concentration of 70% ethanol or DMSO diluted in the culture medium) were tested in parallel to rule out any solvent effects.

### 4.3. Cell Viability Assay

GBM and AST-1 cells (3 × 10^4^ cells/mL) were seeded in 96-well plates, in a final volume of 100 μL/well, and after one day of incubation, CBG (0–50 μM), CBN (0–50 μM), MLT (0–1 mg/mL) alone or in combination were added. Initially, wide concentration ranges were screened for each single agent to determine their individual cytotoxicity profiles. IC_50_ values were calculated using GraphPad Prism 9.0.0 (121) software (GraphPad Software, San Diego, CA, USA). Data were normalized to control values and fitted utilizing the non-linear regression model for log(inhibitor) vs. normalized response-variable slope. Subsequently, a comprehensive combinatorial matrix was performed, evaluating every single dose of each compound against all doses of the other agents. Drug interactions were evaluated using the SynergyFinder platform based on the Bliss independence reference model [49]. This model assumes a multiplicative effect of single drugs as if they acted independently. The nature of the interaction was determined according to the calculated Bliss synergy scores: a score larger than 10 was considered synergistic, a score ranging from −10 to 10 was considered additive, and a score less than −10 was identified as antagonistic. Among these tested combinations, specific concentration ranges were selected for detailed analysis and presented in Figure 2, specifically, for U87 (MLT: 0.2, 0.3, 0.4 mg/mL; CBN: 25, 30, 37.5 μM; CBG: 10, 15, 20 μM), T98 (MLT: 0.5, 0.6, 0.7 mg/mL; CBN: 15, 20 25 μM; CBG: 25, 30, 35 μM), and U251 (MLT: 0.2, 0.4, 0.5 mg/mL; CBN: 20, 25, 30 μM; CBG: 25, 30, 35 μM). These precise dose ranges were chosen because they successfully triggered intermediate-to-high cytotoxic responses while uncovering the most significant synergistic clusters (as shown in the synergy maps), thereby allowing us to identify the optimal therapeutic windows for each distinct genetic background.

Separately, TMZ was evaluated at a single fixed concentration (600 μM) [50], and tested exclusively in combination with synergistic MLT–phytocannabinoid combinations selected for each individual cell line: (U87: MLT 0.3 mg/mL and CBN 25 µM; MLT 0.2 mg/mL and CBG 15 µM. T98: MLT 0.7 mg/mL and CBN 25 µM; MLT 0.6 mg/mL and CBG 30 µM. U251: MLT 0.4 mg/mL and CBN 20 µM; MLT 0.5 mg/mL and CBG 35 µM).

To investigate the potential involvement of ferroptosis, cells were pre-incubated with the specific ferroptosis inhibitor Liproxstatin-1 (Lipro-1, 25–100 nM; Sigma-Aldrich) for 1 h prior to the addition of the single or combined compounds. At least six replicates in each experiment were used for each treatment. After 72 h, cell viability was assessed by adding 0.8 mg/mL of 3-[4,5-dimethylthiazol-2-yl]-2,5 diphenyl tetrazolium bromide (MTT) (Sigma-Aldrich) to the media. The absorbance of samples, solubilized in dimethyl sulfoxide (DMSO), compared to a control (medium only) was measured at 540 nm using the Spectra Max ID3 microplate reader (Molecular Devices, Munich, Germany).

### 4.4. ROS Production

Intracellular oxidative stress levels in GBM cells were evaluated using the fluorescent probe 20,70-dichlorofluorescein (DCFDA, Thermo Fisher Scientific, Segrate, Italy). Cells (5 × 10^4^ cells/mL) were treated with CBG, CBN, MLT alone or in combination for up to 24 h. Hydrogen peroxide 6 µL/mL was used as a positive control. At the end of each incubation period, cells were incubated with DCFDA at 37 °C for 20 min. Quantitative measurements were performed by flow cytometry using a BD Accuri C6 Plus system (BD Biosciences, San Jose, CA, USA) equipped with a 488 nm laser, collecting the signal in the green channel (FL1). For each sample, a minimum of 5000 events were acquired. Debris and doublets were excluded from the analysis based on forward (FSC) and side scatter (SSC) characteristics (FSC-A vs. FSC-H and SSC-A vs. SSC-H). Cells incubated without DCFDA were used as an unstained control to set the baseline autofluorescence gating. Data were analyzed using BD Accuri^TM^ C6 Plus Software Version 1.0.34.1 (BD Biosciences).

### 4.5. Mitochondrial Transmembrane Potential

The mitochondrial transmembrane potential (ΔΨ_m_) was evaluated by 5,5′,6,6′-tetrachloro-1,1′,3,3′-tetraehylbenzimidazolylcarbocyanineiodide (JC1) staining (Thermo Fisher Scientific). GBM cell lines (5 × 10^4^ cells/mL) were seeded into 24-well plates, treated with CBG, CBN, MLT alone or in combination or the vehicle. Subsequently, cells were incubated with JC-1 (10 μg/mL). Carbonyl cyanide chlorophenylhydrazone protonophore (CCCP, 50 μM, Sigma-Aldrich), a mitochondrial uncoupler known to dissipate ΔΨ_m_, was used as a positive control. Quantitative analysis was performed by flow cytometry using a BD Accuri C6 Plus system and related software (BD Biosciences). For each sample, a minimum of 5000 events were acquired. Debris and aggregates were excluded using FSC-A vs. FSC-H gating. JC-1 monomer fluorescence (green) was detected in the FL-1 channel, while JC-1 aggregate fluorescence (red) was detected in the FL-2 channel. Mitochondrial depolarization was expressed as the shift in the ratio of red to green fluorescence intensity.

### 4.6. Determination of Lipid ROS

Intracellular lipid peroxidation and lipid reactive oxygen species (ROS) levels were quantified using the fluorescent probe BODIPY™ 581/591 C11 (Thermo Fisher Scientific) according to the manufacturer’s instructions. Briefly, GBM cells were seeded in 24-well plates at a density of 5 × 10^4^ cells/mL and allowed to adhere. Following 24 h of treatment with MLT, CBN, or CBG alone, or in their respective combinations, the culture medium was removed. The cells were washed with phosphate-buffered saline (PBS) and incubated with 10 μM C11 BODIPY for 30 min at 37 °C in the dark. After incubation, cells were washed twice with PBS, harvested, and immediately analyzed using a BD Accuri C6 Plus flow cytometer equipped with its analysis software (BD Biosciences). For each sample, a minimum of 5000 events were acquired, excluding debris and doublets. To monitor the shifting fluorescence during lipid oxidation, C11 BODIPY was excited at 561 nm to measure the red fluorescence intensity (associated with the unoxidized dye, collected in the red channel) and at 488 nm to measure the green fluorescence intensity (associated with the oxidized dye, collected in the green channel). Lipid peroxidation was expressed as the relative shift in the red/green fluorescence ratio compared to the untreated control group.

### 4.7. Cell Death Assay

GBM cells (5 × 10^4^ cells/mL) were treated with CBG, CBN, MLT alone or in combination for 24 h. Then cells were incubated with 5 μL/well Annexin V-FITC (BD Biosciences) and 2 µg/mL PI (BD Biosciences) for 10 min at 20–25 °C. After washing, fluorescence was analyzed by BD Accuri C6 Plus flow cytometer and its software (BD Biosciences). For each sample, a minimum of 5000 events were collected. Cells were gated on FSC-A versus SSC-A to exclude debris, and doublets were excluded using FSC-A versus FSC-H plots. Annexin V-FITC fluorescence was detected in the green channel, while PI fluorescence was detected in the red channel. Fluorescence compensation was set up using unstained cells, Annexin V-only, and PI-only single-stained controls. Quadrant gating was established based on control samples to distinguish viable (Annexin V−/PI−), early apoptotic (Annexin V+/PI−), late apoptotic (Annexin V+/PI+), and necrotic (Annexin V−/PI+) cells.

### 4.8. Western Blot

Lysates from glioma cells were extracted using a lysis buffer containing a protease-inhibitor cocktail (EuroClone, Milan, Italy). Proteins were separated on 9–12% sodium dodecyl sulphate (SDS)/polyacrylamide gels using a Mini-PROTEAN Tetra Cell system (Bio-Rad, Milan, Italy). Protein transfer to a nitrocellulose membrane was performed using a Mini Trans-Blot Turbo RTA system (Bio-Rad). Non-specific binding sites were blocked with low-fat dry milk (Santa Cruz Biotechnology, Dallas, TX, USA) or bovine serum albumin (BSA) in PBS containing 0.1% Tween 20. Membranes were incubated with phospho-histone H2A.X (#9718, 1:1000; Cell Signaling Technology, Milan, Italy), PARP-1 (sc-8007, 1:500, Santa Cruz Biotechnology), MEL A/B (sc-398788, 1:500, Santa Cruz Biotechnology) and GAPDH (sc-47 724, 1:1000; Santa Cruz Biotechnology) antibodies overnight at 4 °C, followed by incubation with HRP-conjugated secondary antibodies (anti-rabbit antibody, 1:2000; anti-mouse antibody, 1:2000; Cell Signaling Technology). Analysis was performed using LiteAblot PLUS or Turbo kits (EuroClone), ChemiDocTM XRS+ with Image LabTM Software version 6.1.0 (Bio-Rad). GAPDH was used as loading control.

### 4.9. Determination of Mitochondrial Mass

To determine mitochondrial mass by flow cytometry, the MitoBright LT-Green dye was used according to the manufacturer’s instructions (Dojindo Molecular Technologies, Rockville, MD, USA). Briefly, glioma cells seeded in 24-well plates at a density of 5 × 10^4^ cells/mL were treated with CBG, CBN, MLT alone or in combination for 24 h and then incubated with MitoBright LT-Green dye (1:1000) for 30 min at 37 °C in the dark. After washing, cells were analyzed by BD Accuri C6 Plus flow cytometer and its software. For each sample, a minimum of 5000 events were collected. Debris and aggregates were excluded from the analysis. The MitoBright LT-Green signal was excited using a 488 nm laser, and its fluorescence emission was recorded in the green channel. Unstained cells were used as a control to set the baseline autofluorescence gating.

The impairment of mitochondrial mass and integrity was also assessed by COX IV staining. Cells, treated as described above, were fixed in 4% paraformaldehyde and permeabilized with permeabilization solution (1% FBS, 0.1% saponin, 0.1%, sodium azide in PBS). The cells were incubated with mouse anti-human COX IV antibody (1:200, Cell Signaling Technology, Danvers, MA, USA) followed by Alexa fluor 488-conjugated secondary antibody (1:2000, Cell Signaling). Cells were then analyzed by BD Accuri C6 Plus flow cytometer and its software. For each sample, a minimum of 5000 events were acquired. Debris and cell doublets were excluded from the analysis. Alexa Fluor 488 fluorescence was excited by the 488 nm laser and collected in the FL1 channel. Cells stained only with the secondary antibody (or unstained cells) were used as a negative control to define the gating threshold and monitor non-specific binding. Mitochondrial mass alteration was quantified and expressed as the percentage of reduction in COX IV^+^ cells.

### 4.10. Confocal Microscopy

T98 glioma cells (4 × 10^4^/mL) were plated on a µ-Slide 8 Well (Cat. No: 80826, IBIDI, Gräfelfing, Germany) and treated with CBG, CBN, MLT alone or in combination for 24 h. After treatment, cells were fixed 10 min with 2% paraformaldehyde in 0.1% of Tween-20 in PBS followed by 10 min with 4% paraformaldehyde (Sigma-Aldrich) in 0.1% of Tween-20 (Sigma-Aldrich). Then cells were incubated with 5% of BSA and 0.1% of Tween-20 in PBS for 1 h at 20–25 °C. After, cells were labeled with mouse anti-human COX IV antibody (1:200, Cell Signaling Technologies) overnight at 4 °C followed by Alexa fluor 488-conjugated secondary antibody (1:2000, Cell Signaling Technologies) for 1 h at 37 °C. Nuclei were stained with DAPI (Bio-Rad). Slides were analyzed with a C2 Plus confocal microscope (Nikon Europe B.V., Amstelveen, The Netherlands). Magnification = 100×.

### 4.11. Statistical Analysis

GraphPad Prism 9.0.0 (121) software was used for statistical analysis. The results represent the mean ± standard deviation (SD) of three experiments. Data normality and homogeneity of variance were verified using the Shapiro–Wilk test prior to statistical analysis. All datasets followed a normal distribution, justifying the use of parametric analysis. One-way or two-way analysis of variance (ANOVA) was performed followed by Tukey’s multiple comparison test, Dunnett’s comparisons, or Šidák’s comparisons. * *p* < 0.05, ** *p* < 0.01, *** *p* < 0.001, **** *p* < 0.0001. No statistically significant differences were found between untreated (control) with vehicle-treated cells.

## 5. Conclusions

This study evidences a synergistic interaction between minor phytocannabinoids, CBN or CBG, and MLT against GBM. These combined combinations offer a promising alternative to overcome the intrinsic chemoresistance that has long slowed down the clinical progress of standard GBM therapies since the introduction of TMZ. Crucially, the discovery that MLT-CBN and MLT-CBG co-administration selectively triggers cytotoxicity in malignant cells, while exhibiting limited effects on healthy normal astrocytes, provides the first rigorous biochemical validation for clinical observations and case reports. Ultimately, this work shifts the GBM treatment from traditional, evasion-prone apoptotic targeting toward a novel, multi-target death, establishing a foundation for future preclinical testing and targeted translational interventions.

## Data Availability

The original contributions presented in this study are included in the article/Supplementary Materials. Further inquiries can be directed to the corresponding authors.

## Supplementary Materials

The following supporting information can be downloaded at: https://www.mdpi.com/article/10.3390/ijms27156774/s1.

## Abbreviations

The following abbreviations are used in this manuscript:

GBM	Glioblastoma
IDH	Isocitrate Dehydrogenase
CNS	Central Nervous System
TMZ	Temozolomide
MLT	Melatonin
THC	Tetrahydrocannabinol
CBD	Cannabidiol
CBN	Cannabinol
CBG	Cannabigerol
PI	Propidium Iodide
SI	Selectivity Index
MFI	Mean Fluorescence Intensity
γH2AX	Phosphorylated Form of the Histone γH2AX
SD	Standard Deviation
ROS	Reactive Oxygen Species
DCFDA	20,70-Dichlorofluorescein
Δψ_m_	Mitochondrial Membrane Potential
JC1	5,5′,6,6′-Tetrachloro-1,1′,3,3′-tetraehylbenzimidazolylcarbocyanineiodide
COX-IV	COX Subunit IV
MTT	3-[4,5-Dimethylthiazol-2-yl]-2,5 Diphenyl Tetrazolium Bromide
DMSO	Dimethyl Sulfoxide
CCCP	Carbonyl Cyanide Chlorophenylhydrazone Protonophore
PBS	Phosphate-Buffered Saline
DAPI	Diamidino-2-phenylindole
EGF	Epidermal Growth Factor
HGF	Hepatocyte Growth Factor
IGF	Insulin-like Growth Factor
SDS	Sodium dodecyl sulphate
BSA	Bovine Serum Albumin
PARP 1	Poly (ADP-ribose) Polymerase 1
BBB	Blood–Brain Barrier
IC_50_	Compound Concentration Required to Inhibit 50% of Cell Viability
BCS	Biopharmaceutics Classification System
