# Supplementary material for: Synergistic In Vitro Effects of Minor Phytocannabinoids and Melatonin Combinations Against Human Glioblastoma Cells
Source: Int J Mol Sci. 2026 Jul 29;27(15):6774. doi: 10.3390/ijms27156774 (PMC13466516; doi:10.3390/ijms27156774)
Supplement: Supplementary file 1 [file ijms-27-06774-s001.zip › Supplementary Figures.pdf]

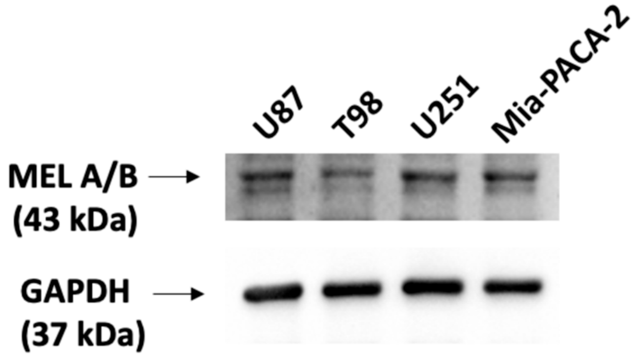


**Figure S1.** Western blot analysis of MEL A/B protein expression. Expression levels of MEL A/B (predicted molecular weight of 43 kDa) were evaluated in U87, T98, and U251 glioblastoma cells, alongside Mia-PACA-2 pancreatic cancer cells used as positive control [24]. GAPDH (37 kDa) was utilized as an internal loading control. Blots are representative of three independent biological experiments.

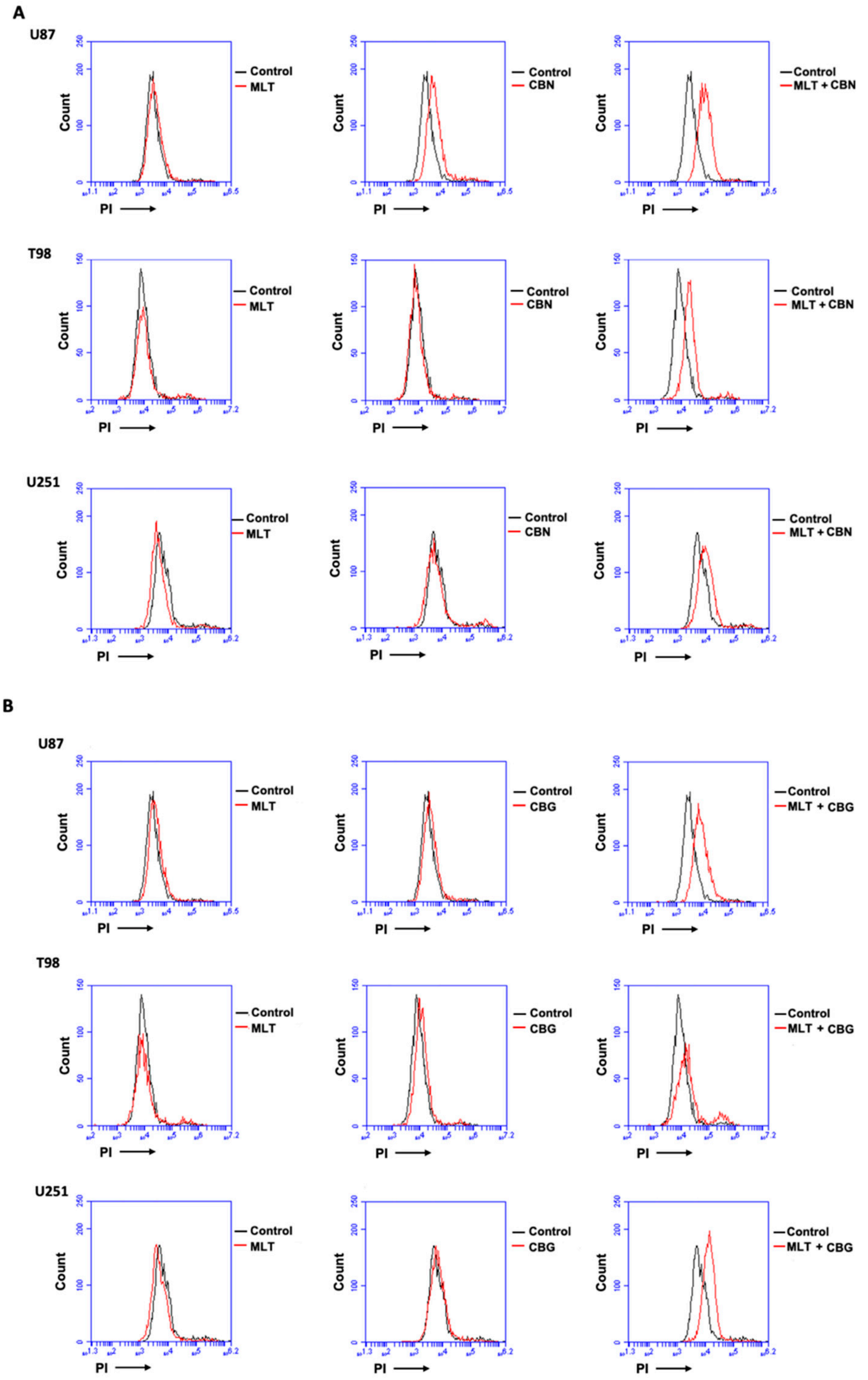


**Figure S2.** Flow cytometric analysis of cell death in glioblastoma cell lines following single and combined treatments. U87, T98, and U251 cell lines were treated with MLT, CBN, CBG, or their respective combinations for 24 h (U87: MLT 0.3 mg/ml and CBN 25 μM; MLT 0.2 mg/ml and CBG 15 μM. T98: MLT 0.7 mg/ml and CBN 25 μM; MLT 0.6 mg/ml and CBG 30 μM. U251: MLT 0.4 mg/ml and CBN 20 μM; MLT 0.5 mg/ml and CBG 35 μM). Cell death were assessed via Propidium Iodide (PI) staining and flow cytometry. (A) Representative flow cytometry histograms for U87, T98, and U251 lines under single or combined MLT and CBN (A) exposure. (B) Representative flow cytometry histograms for the same GBM lines under single or combined MLT and CBG exposure (B).

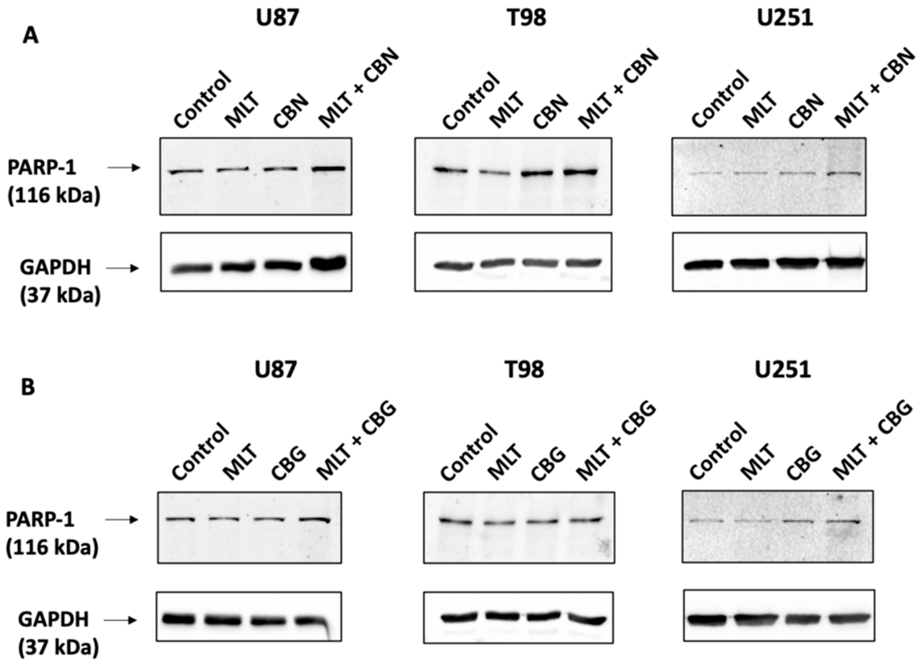


**Figure S3.** Effect of melatonin and cannabinoids combinations on PARP-1 expression in GBM cells. Representative Western blot bands showing protein levels of PARP-1 (116 kDa) and GAPDH (37 kDa), used as the internal loading control. A) Cells treated with MLT, CBN or their respective combinations for 24 h (U87: MLT 0.3 mg/ml and CBN 25 μM. T98: MLT 0.7 mg/ml and CBN 25 μM. U251: MLT 0.4 mg/ml and CBN 20 μM). B) Cells treated with MLT, CBG or their respective combinations for 24 h (U87: MLT 0.2 mg/ml and CBG 15 μM. T98: MLT 0.6 mg/ml and CBG 30 μM. U251: MLT 0.5 mg/ml and CBG 35 μM). Blots are representative of three independent biological experiments.

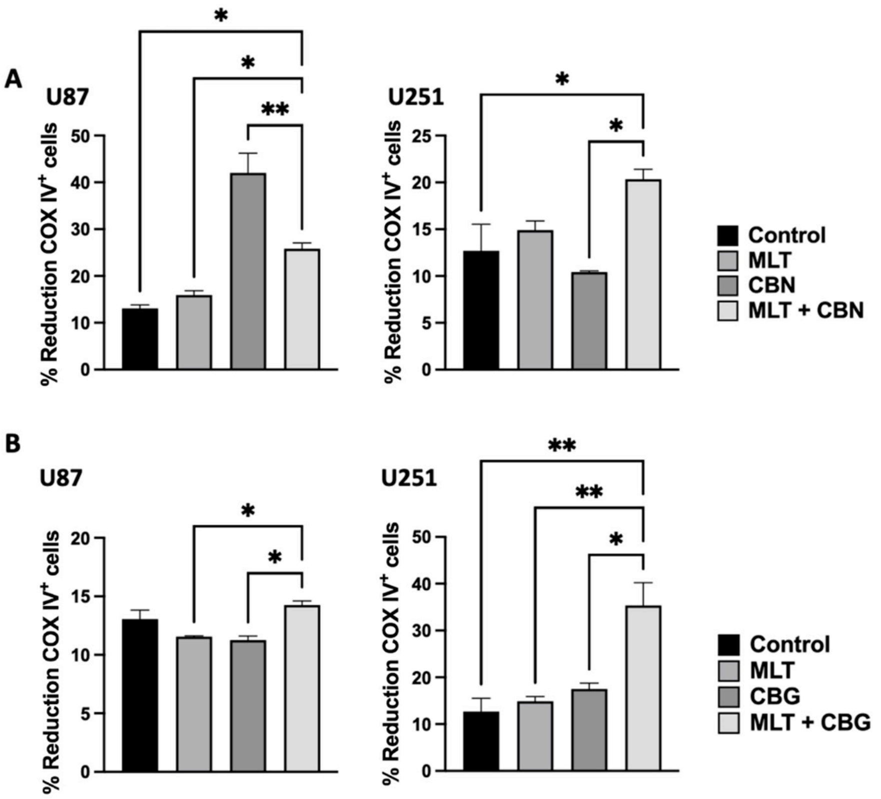


**Figure S4.** Flow cytometric evaluation of COX-IV expression in treated U87 and U251 cells. Flow cytometric analysis of Cytochrome c Oxidase subunit IV (COX-IV) expression (MFI, fold change) in U87 and U251 cell line under the respective single or combined treatments (U87: MLT 0.3 mg/ml and CBN 25 μM; MLT 0.2 mg/ml and CBG 15 μM. U251: MLT 0.4 mg/ml and CBN 20 μM; MLT 0.5 mg/ml and CBG 35 μM). Bar graphs indicate the percentage reduction of COX-IV positive cells. Data are presented as mean ± SD of at least three independent biological replicates. Statistical significance was determined using a one-way ANOVA followed by Tukey's post-hoc test for multiple comparisons (* $p < 0.05$, ** $p < 0.01$, *** $p < 0.001$, **** $p < 0.0001$).

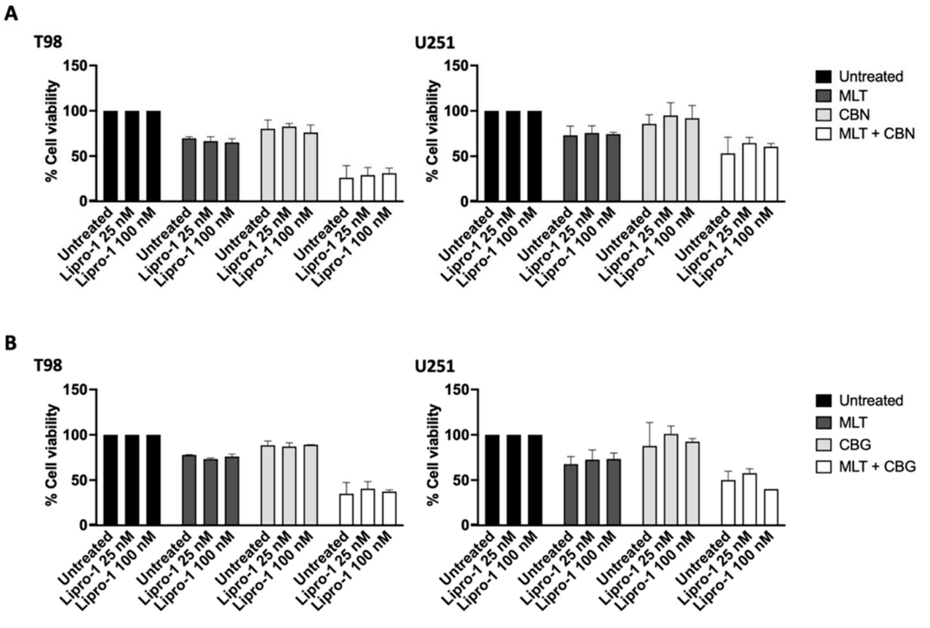


Figure S5. Pharmacological inhibition of ferroptosis does not rescue cannabinoid- and MLT-induced cytotoxicity in glioblastoma cells. Cell viability was assessed by MTT assay in T98, and U251 cells after 72 h of exposure to single or combined treatments (U87: MLT 0.3 mg/ml and CBN 25 μM; MLT 0.2 mg/ml and CBG 15 μM. T98: MLT 0.7 mg/ml and CBN 25 μM; MLT 0.6 mg/ml and CBG 30 μM. U251: MLT 0.4 mg/ml and CBN 20 μM; MLT 0.5 mg/ml and CBG 35 μM) in the presence or absence of the specific ferroptosis inhibitor Liproxstatin-1 (Lipro-1) at 25 nM and 100 nM. (A) Effects of Lipro-1 pre-treatment on cell viability under single MLT or CBN exposure and their combined formulation (MLT + CBN). (B) Effects of Lipro-1 pre-treatment on cell viability under single MLT or CBG exposure and their combined formulation (MLT + CBG). Data are presented as mean ± SD of at least three independent biological replicates. Statistical significance was determined using a two-way ANOVA followed by Dunnett's post-hoc test for multiple comparisons. Statistical analysis showed no significant differences between groups treated with the compounds alone and those pre-incubated with Lipro-1.
